# Genetic Structure and Evolutionary History of *Rhinopithecus roxellana* in Qinling Mountains, Central China

**DOI:** 10.3389/fgene.2020.611914

**Published:** 2021-01-20

**Authors:** Yuli Li, Kang Huang, Shiyi Tang, Li Feng, Jia Yang, Zhonghu Li, Baoguo Li

**Affiliations:** ^1^Shaanxi Key Laboratory for Animal Conservation, College of Life Sciences, Northwest University, Xi'an, China; ^2^School of Pharmacy, Xi'an Jiaotong University, Xi'an, China; ^3^Key Laboratory of Resource Biology and Biotechnology in Western China, Ministry of Education, College of Life Sciences, Northwest University, Xi'an, China; ^4^Center for Excellence in Animal Evolution and Genetics, Chinese Academy of Sciences, Kunming, China

**Keywords:** *Rhinopithecus roxellana*, population structure, gene flow, ecological niche models, evolutionary history

## Abstract

The Qinling mountainous region is one of the world's biodiversity hotspots and provides refuges for many endangered endemic animals. The golden snub-nosed monkeys (*Rhinopithecus roxellana*) are considered as a flagship species in this area. Here, we depicted the genetic structure and evolutionary history via microsatellite markers and combination with the ecological niche models (ENMs) to elucidate the intraspecific divergent and the impacts of the population demography on our focal species. Our results revealed three distinct subpopulations of *R. roxellana* and also uncovered asymmetric historical and symmetric contemporary gene flow that existed. Our evolutionary dynamics analyses based on diyabc suggested that the intraspecific divergence accompanied with effective population sizes changes. The ENM result implied that the distribution range of this species experienced expansion during the last glacial maximum (LGM). Our results highlighted that geological factors could contribute to the high genetic differentiation within the *R. roxellana* in the Qinling Mountains. We also provided a new insight into conservation management plans with endangered species in this region.

## Background

Historical processes such as geographic changes and human activities have greatly affected patterns of dispersal and genetic migration among populations; these processes could restrict gene flow and accelerate genetic differentiation through inbreeding and genetic drift (Waage and Greathead, 1988; Frankham, 2005) and consequently shape population structure and evolutionary history (Balkenhol et al., 2009). In many species, empirical studies have indicated that mountains (Lait and Burg, 2013), rivers (Chambers and Garant, 2010), and deserts (Astrid et al., 2012) could act as physical or ecological obstacles to prevent animal dispersal to mitigate gene flow. In addition, as humans continue to expand across the world, many once-continuous landscapes become divided into separate fragmented patches (Hanski, 1994; Newbold et al., 2015). Low genetic variations will decrease the adaptive abilities of species to circumstances, and then this species become more vulnerable when environmental conditions changes, which will finally lead to their extinction (Frankham, 2005; Willi et al., 2006; Burkey, 2010).

The golden snub-nosed monkey, *Rhinopithecus roxellana*, is an Asian colobine endemic to the temperate forests of the mountainous regions in central China (Li et al., 2002; Kirkpatrick and Grueter, 2010), which has a typical multi-level social structure discovered in primate (Kirkpatrick and Grueter, 2010). The society of this monkey consists of four levels: unit, band, herd, and troop (Grueter et al., 2012). Furthermore, the bands can be classified into the breeding band (BB) and the all-male band (AMB) (Kirkpatrick and Grueter, 2010; Grueter et al., 2012). This primate once ranged widely across southern, southwestern, and central China (Li et al., 2003). However, due to the historical changes and continuous expansion of human populations and their corresponding activities, numbers and ranges of *R. roxellana* have been significantly reduced. In consequence, this primate today only distribute within the three isolated areas among four provinces (Shaanxi, Sichuan, Gansu, and Hubei), and its current estimated population size is no more than 20,000 individuals (Ren et al., 1998; Li et al., 2002, 2007).

**Figure 1:**
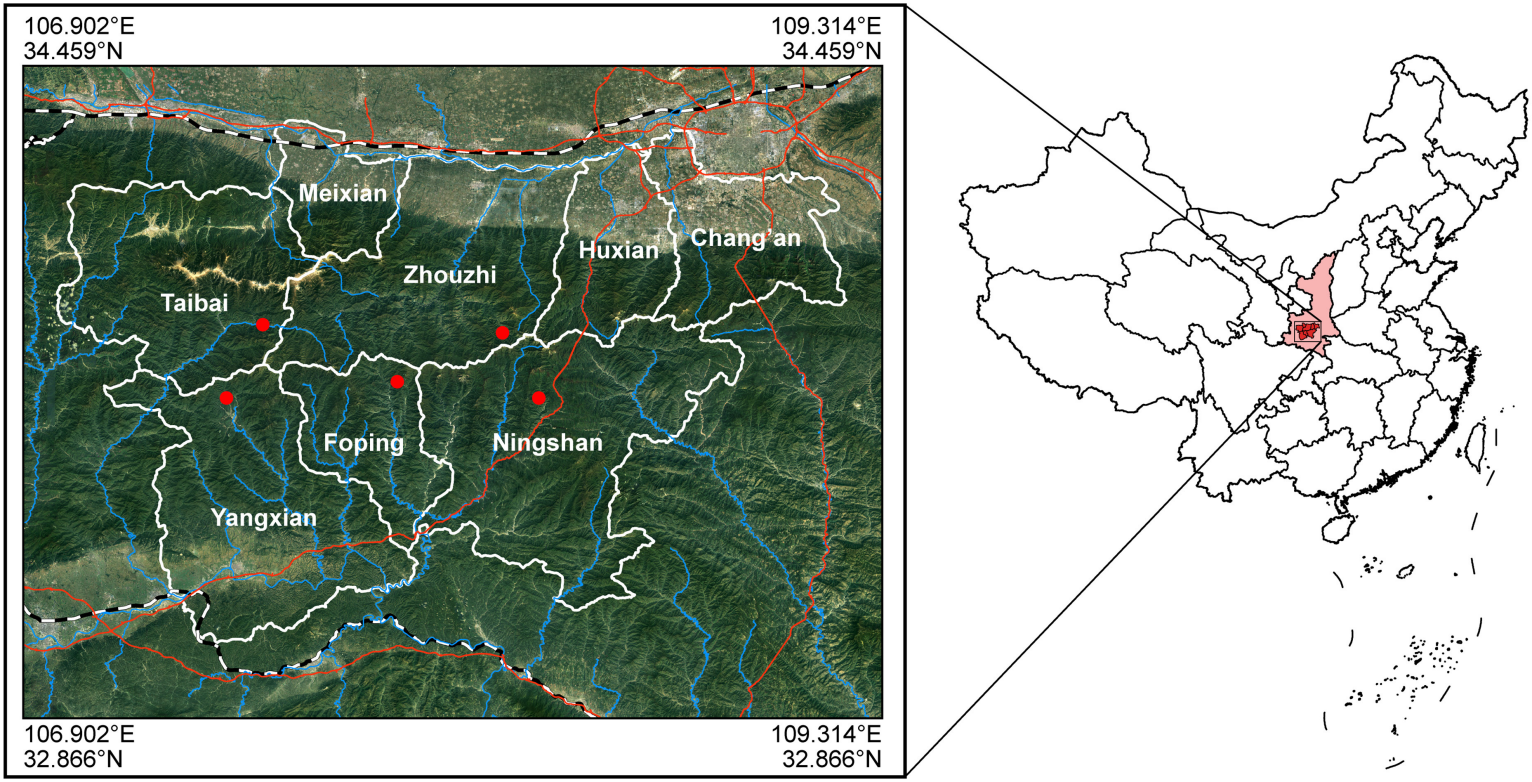
Sampling sites: the range of coordinates were 107.52-108.38°E, 33.64-33.82°N.

In Shaanxi, *R. roxellana* occurs only in the Qinling Mountains (Li et al., 2000; Wang et al., 2014). This area is a major biodiversity hotspot of China with many unique and rare plant and/or animal species (Norton et al., 2011; Zhang et al., 2016). Within the mountains, 39 *R. roxellana* troops comprising a total of 4,000 individuals are distributed across the counties of Zhouzhi, Ningshan, Yangxian, Taibai, and Foping (Figure 1; Li et al., 2000). However, during the past few years, land reclamation, tree-felling, illegal hunting, and infrastructural development have all posed critical threats to the habitats of golden snub-nosed monkeys in these regions (Feng et al., 2009; Zhang et al., 2016). Previous research has shown that expanding human impact in nearby the Qinling Mountains has resulted in local extinction and population decline of *R. roxellana* (Li et al., 2002; Wang et al., 2014).

Based on the microsatellite data, Pan et al. (2005) showed the *R. roxellana* had relatively high intraspecific genetic diversity and formed different genetic structures at fine scale in China, e.g., the Minshan Mountains, the Shennongjia Mountains, and the Qinling Mountains. Also, Huang et al. (2016a) had detected that the genetic diversity and effective population size of *R. roxellana* in Qinling Mountains were not significantly decreased, which is in conflict with their expectations. In addition, a recent study has that, even under threat of habitat fragmentation, internal adjustment mechanisms in multilevel social systems can effectively avoid inbreeding due to the bridging role of certain social units (non-breeding groups: AMB) at a small scale within the Qinling Mountains (Li et al., 2020). However, the evolutionary history and demography of the *R. roxellana* inhabiting the Qinling Mountains remain poorly understood (Pan et al., 2005).

In this study, using microsatellite data, we evaluate the genetic diversity and gene flow within the Qinling populations and also investigated its complicated evolutionary history. We attempt to figure out the following: (i) whether the golden snub-nosed monkey was threatened by genetic homogeneity in fragmented habitat and, if so, (ii) whether the historical shifts facilitated the current genetic differentiation within *R. roxellana*, and (iii) whether and how the gene flow impacted its genetic structure.

## Methods

### Genetic Sampling

We collected a total of 344 fecal and hair samples of five representative populations scattered among five counties (Zhouzhi, Foping, Taibai, Yangxian, and Ningshan), which spread over five National Nature Reserve [the Zhouzhi National Nature Reserve (ZNNR), the Foping National Nature Reserve (FNNR), the Taibaishan National Nature Reserve (TNNR), the Changqing Natioanl Nature Reserve (CNNR), and Ningshan Nature Reserve (NNR); Figure 1: 107.52-108.38°E, 33.64-33.82°N]. These regions have semi-humid montane climate, and their elevations range from 1,100 to 2,930 m above sea level (Li et al., 2000, 2003). The average annual temperature of these areas is 8.59°C, with a minimum temperature in January (−17.7°C) and a maximum temperature in August (34.6°C). The average rainfall per annum is 577.64 mm. Details of the sample information are presented in Table 1; sampling locations are shown in Figure 1.

**Table 1:** Genetic diversities of five populations.

**Band**	**Reserve**	**County**	**Longitude (^**°**^E)**	**Latitude (^**°**^N)**	**Population size**	**Sample size**	***F*_**IS**_**	**Genetic diversity**				
								***A*_**O**_**	***H*_**O**_**	***H*_**E**_**	**PIC**	***A*_**R**_**
GNG	ZNNR	Zhouzhi	108.28	33.8	173	84	0.009	4.059	0.563	0.558	0.508	2.497
DPY	FNNR	Foping	107.99	33.68	133	71	0.081	5.353	0.566	0.629	0.473	2.342
SZZ	CNNR	Yangxian	107.52	33.64	142	67	0.065	5.765	0.607	0.650	0.538	2.642
HTP	TNNR	Tabai	107.62	33.82	118	62	0.095	5.176	0.614	0.672	0.527	2.539
CYG	NNR	Ningshan	108.38	33.64	72	33	0.031	4.882	0.624	0.605	0.510	2.532

*F_IS_ denotes the Wright's inbreeding coefficient*.

*Six genetic diversity indices were presented, i.e., number of alleles (A_O_), observed and expected heterozygosity (H_O_ and H_E_, respectively), polymorphism information content (PIC), and allelic richness (A_R_)*.

Because our focal species inhabit mountainous highlands and shy away from humans, it is quite hard to follow them across steep cliffs and precipitous valleys; it took us more than 1 year to collect the samples from March 2016 to April 2017. The percentage of fecal samples was 90 in our all samples, and they were stored in DETs [20% dimethyl sulfoxide (DMSO), 0.25 M of sodium-EDTA, 100 mM of Tris·HCl, pH 7.5, and NaCl to saturation] solution at −20°C (Allen et al., 1998). The hair samples, collected with a stick with adhesive tape by glue and fruit bait on an 80 × 6 cm wooden board, were mainly obtained from the Zhouzhi BB (because this band was half-habituated for 20 years on field observation) and then stored in silica gel for drying at room temperature.

### Molecular Methods

Follicle DNA was extracted with proteinase *K* digestion in a PCR-compatible buffer, while fecal DNA was extracted using QIAamp DNA Stool Mini Kits (Qiagen, German). All samples were amplified based on the primers of 19 tetra-nucleotide microsatellite loci (see Supplementary Table 1 for locus profile) in an ABI Veriti Thermal Cycler with the following protocol: 95°C for 5 min, followed by 30 cycles (94°C for 30 s, 55–60°C for 45 s, 72°C for 45 s), and 72°C for 10 min. PCR products were segregated with an ABI PRISM 3100 Genetic Analyser, and their sizes relative to the internal size standard (ROX-labeled HD400) were determined using genemapper V3.7 (Applied Biosystems). To prevent genotyping errors such as false allele and allelic dropout, homozygote genotypes were confirmed by five independent replicates, with all heterozygotes observed and confirmed by at least three separate reactions (Taberlet et al., 1996). Replicates were detected by polyrelatedness V1.6 (Huang et al., 2016b) and excluded from before subsequent analyses.

### Genetic Diversity

We used microchecker v2.2.3 to test the presence of null alleles for all loci (van Oosterhout et al., 2010). For each population, we calculated the genetic diversity indices that included number of alleles (*A*_O_), observed heterozygosity (*H*_O_), expected heterozygosity (*H*_E_), polymorphic information content (PIC), allelic richness (*A*_R_), and Wright's inbreeding coefficient at each locus utilizing genaiex V6.5 (Peakall and Smouse, 2012). We performed a Hardy–Weinberg equilibrium (HWE) test for each band at each locus using Fisher's exact tests in genepop V4.3 (Rousset, 2008). Significance thresholds were adjusted for multiple tests by the sequential Bonferroni procedure (Rice, 1989).

We employed two methods to test the presence of bottleneck effects within different populations. The first method was based on deviations of allele frequencies in calculations of heterozygosity, where we used the signed test and two-tailed Wilcoxon test in bottleneck V1.2.02 (Piry et al., 1999). We considered two types of mutation models: (i) a two-phase model (*TPM*) with 95% stepwise mutations and a variance of 12 and (ii) a stepwise mutation model (*SMM*) with iteration number set to 1,000. The second method involved calculation of the *GW* coefficient in arlequin V3.6 (Excoffier and Lischer, 2010).

### Population Differentiation and Structure

Genetic differentiation among populations was evaluated using θ (*F*_ST_) across loci with the 100,000 permutations with arlequin V3.6 (Excoffier and Lischer, 2010). The number of steps in the Markov chain was set 100,000, and that of burn-in steps was 10,000. Moreover, we performed AMOVA analysis in arlequin V3.6 (Excoffier and Lischer, 2010) to estimate genetic variations among the populations. The significance of fixation indices was tested using 10,000 permutations.

Bayesian clustering was performed using structure V2.3.4 (Pritchard et al., 2000) to examine the population genetic structure. We first assessed the occurrence of population subdivision under an admixture model and with allele frequencies correlated. The program was run for *K* from 1 to 10. For each run, we used 1,000,000 *MCMC* cycles following 500,000 burn-in cycles. Two alternative methods were utilized to estimate the most likely number (*K*) of genetic clusters with the program structure harvster (Earl and Vonholdt, 2012, i.e., by tracing the change in the average of log-likelihood *L*(*K*) (Pritchard et al., 2000) and by calculating Δ*K* (Evanno et al., 2005).

### Genetic Migration Analyses

To explore the historical genetic gene flow of *Rhinopithecus roxellana* in the Qinling Mountains, we used the program migrate-n v3.6 (Beerli, 2006) to assess pairwise estimates of migration rates (*N*m) among the five populations based on the microsatellite datasets. We performed maximum-likelihood analyses in migrate-n v 3.6 using three long chains (100,000 runs) and 10 short chains (10,000 runs), and the burn-in was set as 10,000. We repeated this procedure five times to obtain the average maximum-likelihood estimates with 95% confidence intervals (CIs).

Moreover, in order to obtain the contemporary genetic migration among intraspecific lineages, we employed bayesass v3.0 (Wilson and Rannala, 2002) to calculate the intra-population migration rates. We conducted the analyses with burn-in of 1,000,000 iterations, setting 1,000 as the sampling frequency. Ten independent runs were executed to seek the model convergence.

### Demographic History

**Figure 2:**
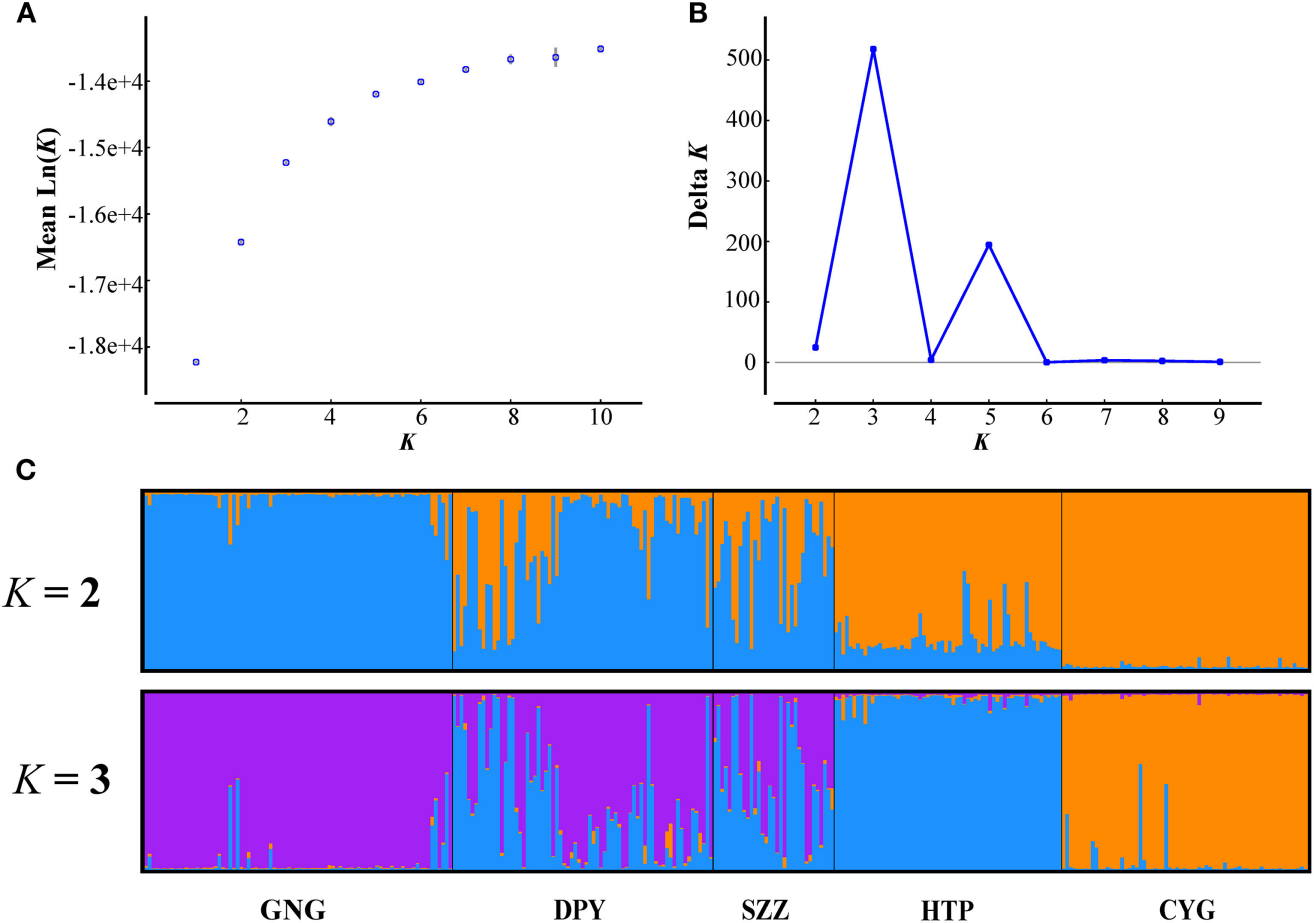
Structure analyses using the locprior model. **(A,B)** The mean estimated *L*(*K*) and Δ*K* as a function of number of clusters (*K*). **(C)** The bar plots for *K* = 2 and 3.

**Figure 3:**
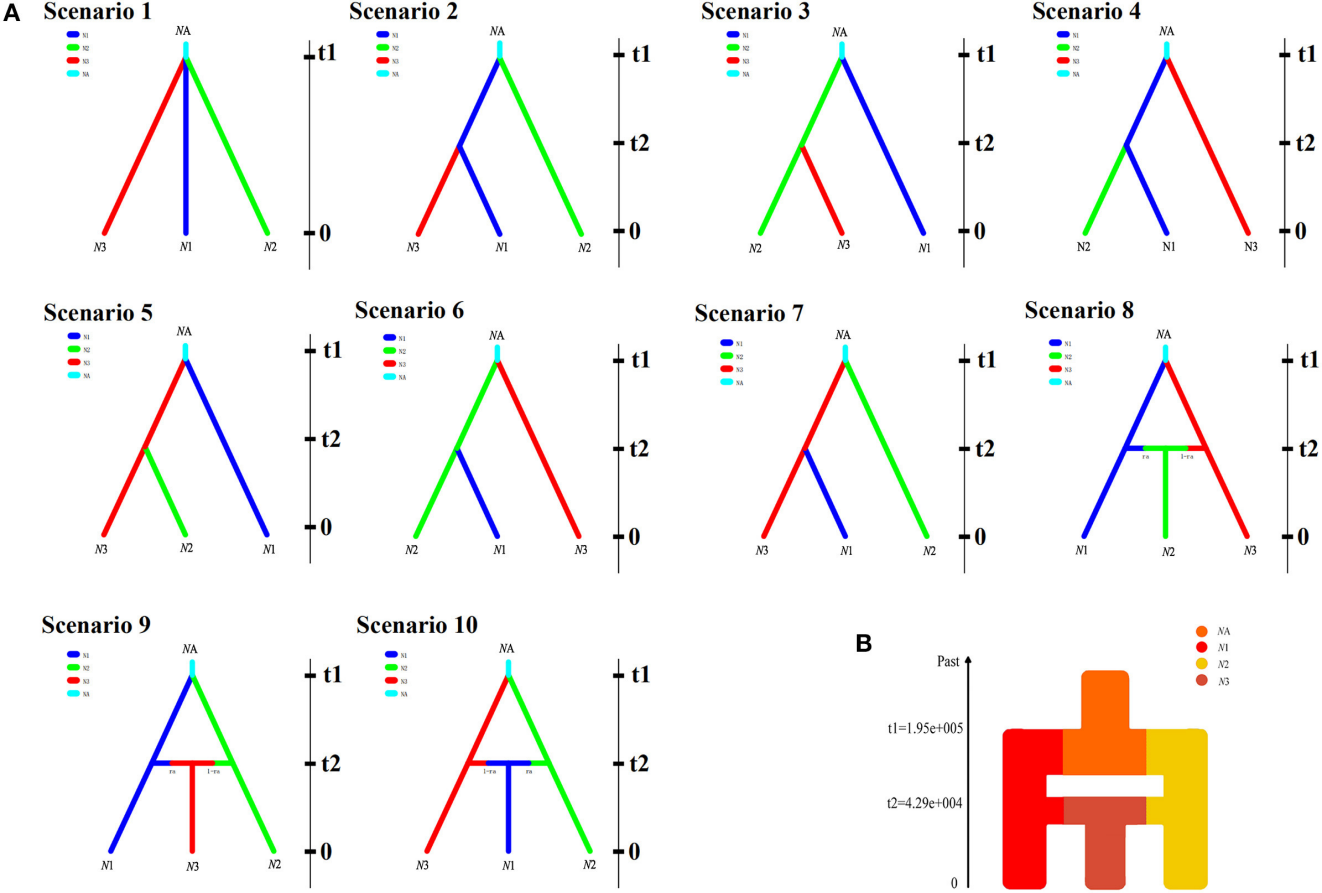
**(A)** The 10 scenarios of population history of five populations in *Rhinopithecus roxellana* in Qinling Mountains with diyabc v 2.04. *N*1, *N*2, and *N*3 denote the current population lineages, while *N*A represents the ancestral population lineage. t1 and t2 are divergence times for the depicted event. **(B)** Schematic representation of changes in population size tested within the three population lineages.

In order to explore the plausible scenarios of divergence and population dynamics within *R. roxellana*, approximate Bayesian computation (ABC) was used in diyabc v 2.04 (Cornuet et al., 2014). Based on the structure results (Figure 2), 10 historical population divergence scenarios for these lineages were simulated by diyabc analysis (Supplementary Table 2). We assumed uniform priors on all parameters, and then we used a goodness-of-fit test to check the priors of all parameters before implementing the simulations (Figure 3A). To estimate the divergence times among the lineages, the average generation time of *R. roxellana* was assumed to be 5 years, following Oleksyk et al. (2010).

### Ecological Niche Modeling

Ecological niche modeling (ENM) analyses were performed with maxent v3.3.3k (Phillips et al., 2006) to assess the ecological niche of each lineage and to predict their potential range based on their georeferenced localities and environmental variables thereof. Only wild occurrences have been taken into account for the ENM analyses. The occurrence data of *R. roxellana* were obtained from our field observations, literature (Wen and Wen, 2006; Wen, 2009), and the records from two sources: the Global Biodiversity Information Facility (GBIF; https://www.gbif.org/) and the National Specimen Information Infrastructure (NSII; www.nsii.org.cn). In total, 131 georeferenced points were obtained.

We obtained 19 bioclimatic variables at 2.5 arc-minute resolutions from WorldClim (www.worldclim.org) (Hijmans et al., 2005) for four periods: the current, the Holocene (HOL; 12 ka–current), the last glacial maximum (LGM; 18–21 ka), and the last interglacial period (LIG; 120–140 ka). To avoid model over-fitting linked to correlated climatic parameters, only those seven variables that had low correlation coefficients with one another (*r* < 0.8) were retained for subsequent analyses (Supplementary Table 3). All ecological distribution models were visualized in arcgis 10.5.

## Results

### Genetic Diversity

A total of 361 samples were collected. After exclusion of 44 repeated samples as determined by microsatellite profiles, we eventually established a dataset consisting 317 individuals. With the use of micro-checker, the frequencies of null alleles at each of the 19 loci were found to be lower than the threshold frequency (*v* = 0.15) across the five populations. The location, population size, and sample size of each band are presented in Table 1.

The genetic diversity indices of each band are also presented in Table 1. On the whole, genetic variability was moderate at the population level. The number of alleles per locus ranged from 4 to 6, averaging 5.047; observed heterozygosity (*H*_O_) ranged from 0.563 to 0.624, averaging 0.595; expected heterozygosity (*H*_E_) was from 0.558 to 0.672, averaging 0.623; the polymorphism information content (PIC) was from 0.473 to 0.538, averaging 0.511; and allelic richness ranged from 2.342 to 2.642, averaging 2.511 for the populations. The values of Wright's inbreeding coefficient (*F*_IS_) showed that the effect of inbreeding to five populations was weak.

No evidence of bottleneck effects has been found from the microsatellite data for each population. The results of bottleneck effects tests are shown in Table 2; none of the sign or Wilcoxon tests suggest heterozygosity excess or deficiency in either the SMM or TPM mutation models. The lowest *P*-value was 0.060 (Wilcoxon test under TPM model in DPY). The *GW* coefficients of all bands were above the empirical value 0.132 (Garza and Williamson, 2001), and the lowest *GW* coefficient was 0.846 ± 0.177 (CYG).

**Table 2:** Bottleneck effect tests of five populations.

**Band**	**Sign text**		**Wilcoxon test**		***M* ± SD**
	***TPM***	***SMM***	***TPM***	***SMM***	
GNG	0.528	0.076	0.865	0.325	0.880 ± 0.147
DPY	0.129	0.277	0.060	0.156	0.859 ± 0.164
SZZ	0.353	0.359	0.244	0.678	0.849 ± 0.197
HTP	0.430	0.428	0.329	0.644	0.858 ± 0.142
CYG	0.193	0.206	0.064	0.132	0.846 ± 0.177

*No results were significant*.

*TPM denotes two-phase model, SMM denotes step-wise mutation model, and M is the Garza and Williamson (2001) coefficient*.

### Population Differentiation and Structure

The results of *F*_ST_ and permutation test revealed a relatively low but significant genetic divergence among those five bands (*P* < 0.05, Table 3). The AMOVA analyses showed that genetic variation among and within five populations was 15.74 and 84.26%, respectively (Table 4). According to the structure analysis of microsatellite data, the most likely number of genetic clusters was at 3, and the scenario of *K* = 2 was also given for comparison. The *L*(*K*) (the probability of the data given *K* and the model) and Δ*K* (using the method of Evanno et al., 2005) as a function of selecting most likely *K-*value, and the bar plot of assignment matrix of locprior analysis are shown in Figure 2. Monkeys from three populations (e.g., GNG, DPY, and SZZ) were collectively classified into a single cluster, while the remaining two monkey populations, HTP and CYG, were each assigned to a separate cluster.

**Table 3:** Pairwise *F*_ST_ (lower triangular) and permutation test.

	**GNG**	**DPY**	**SZZ**	**HTP**
DPY	0.019^*^			
SZZ	0.030^*^	0.034^*^		
HTP	0.039^*^	0.037^*^	0.039^*^	
CYG	0.083^*^	0.066^*^	0.077^*^	0.051^*^

* *P < 0.05*.

**Table 4:** Results of AMOVA.

**Source of variation**	***d*. *f*.**	**Sum of squares**	**Variance components**	**Variation %**
Among populations	4	547.705	1.05401 *Va*	15.74
Within populations	629	3547.872	5.64050 *Vb*	84.26
Total	633	4095.577	6.69451	

### Gene Flow

Our study revealed asymmetrical historical gene flow among the five populations by migrate, with the greatest gene migration between CYG and DPY (54.1218; Table 5) and the lowest between GNG and CYG (6.8760; Table 5). Moreover, bayesass analysis revealed contemporary genetic migration was symmetrical among the 10 related pairs. Estimated population sizes according to Bayesian modes, with 95% CI, were 0.3074 (95% CI: 0.2936–0.3661) for GNG, 0.4262 (95% CI: 0.4038–0.4502) for DPY, 0.2567 (95% CI: 0.2427–0.2719) for SZZ, 0.6696 (95% CI: 0.6341–0.7078) for HTP, and 1.4484 (95% CI: 1.3051–1.5423) for CYG.

**Table 5:** Rates of contemporary and historical gene flows among five populations using microsatellite data with the programs bayesass, migrate, and bidirectional gene flow (*N*m = immigrants per generation).

	**GNG**	**DPY**	**SZZ**	**HTP**	**CYG**
**Bayesass**					
GNG	–	0.0045 (0.0013–0.0094)	0.0038 (0–0.0078)	0.0038 (0.0017–0.0077)	0.0041 (0.0023–0.0060)
DPY	0.0097 (0.0054–0.0176)	–	0.0056 (0.0021–0.0084)	0.0044 (0–0.0143)	0.0053 (0.0027–0.0092)
SZZ	0.0051 (0.0022–0.0105)	0.0055 (0.0009–0.0094)	–	0.0050 (0–0.0117)	0.0072 (0.0014–0.0128)
HTP	0.0046 (0–0.0096)	0.0046 (0.0023–0.0075)	0.0046 (0–0.0089)	–	0.0047 (0.0018–0.0077)
CYG	0.0093 (0–0.0197)	0.0091 (0.0030–0.0176)	0.0023 (0–0.0067)	0.0056 (0–0.0083)	–
**Migrate**					
GNG	–	17.2104 (15.6694–18.8496)	26.1928 (24.2800–28.2037)	24.6458 (22.7858–26.6049)	12.963 (11.6314–14.3922)
DPY	20.1345 (18.6601–21.6870)	–	26.7072 (25.0050–28.4849)	17.1630 (15.8051–18.5953)	54.1218 (51.6823–26.6378)
SZZ	18.7277 (17.1183–20.4326)	38.0089 (21.4185–25.1390)	–	23.2310 (21.4185–25.1390)	26.3535 (24.4257–28.3757)
HTP	13.7162 (12.6706–14.8180)	16.3020 (15.1622–17.4970)	13.9972 (12.9361–15.1145)	–	9.8459 (8.9673–10.7804)
CYG	6.8760 (6.2652–7.5248)	26.0904 (24.8841–27.3354)	9.8011 (9.0647–10.5769)	8.6441 (7.9570–9.3706)	–
***N*****m**					
GNG	–	10.5810 (8.3546–12.6872)	16.1033 (14.8424–18.437)	15.1522 (13.6046–17.7099)	7.9697 (5.4052–9.8011)
DPY	17.1626 (15.0317–19.2484)	–	22.7652 (20.4299–24.5679)	14.6297 (12.6118–16.1843)	16.1334 (14.1217–18.3535)
SZZ	19.6148 (17.3025–21.5698)	19.5138 (17.2316–21.9637)	–	11.9268 (9.2373–13.9993)	13.5299 (11.3314–15.3275)
HTP	18.3687 (16.0359–20.6790)	21.8316 (19.117–23. 4606)	18.7451 (16. 998–20.5518)	–	13.1856 (11. 221–15.4657)
CYG	19.4866 (17.3668–21.6047)	13.9402 (11.2919–15.3879)	17.7763 (15.0038–19.0291)	14.4974 (12.4778–17.1034)	–

*95% confidence intervals are presented in parentheses*.

### Evolutionary Dynamics History

In the diyabc analysis, the posterior probability for scenario 8 (with 95% CI) was 0.914 (95% CI: 0.906–0.942), much higher than that of the other nine scenarios (Supplementary Figure 1). Our ABC demographic analysis detected that the first divergence in our focal species formed *N*1 and *N*3 lineages and the second divergence formed *N*2, which originated from the second contact of the *N*1 and *N*3 lineages (Figure 3B). The median values of the effective population sizes of *N*1, *N*2, and *N*3 were 2.18 × 10^5^, 1.69 × 10^5^, and 1.44 × 10^5^, respectively, whereas *N*A was 1.85 × 10^6^ (Table 6). In addition, the mean values of effective populations of *N*1, *N*2, and *N*3 were 2.61 × 10^5^, 2.07 × 10^5^, and 1.75 × 10^5^, respectively, whereas *N*A was 1.96 × 10^6^ (Table 6). The estimated median time of divergence between *N*1 (GNG, DPY, and SZZ populations) and *N*3 (CYG populations) (t1) from the common ancestor was 1.95 × 10^5^ generations ago, *N*2 (HTP populations) diverged from the *N*1, and *N*3 (t2) was 4.51 × 10^4^ generations ago (Table 6).

**Table 6:** Demographic approximate Bayesian computation (ABC) models for *Rhinopithecus roxellana* in Qinling Mountains.

**Parameters**	***N*1**	***N*2**	***N*3**	***N*A**	**t1 (generations)**	**t2 (generations)**	**μ**	***P***
Median	2.18E+005	1.69E+005	1.44E+005	1.85E+006	1.34E+005	4.51E+004	1.92E−006	0.5
Mean	2.61E+005	2.07E+005	1.75E+005	1.96E+006	1.95E+005	4.29E+004	2.22E−006	0.5
Lower bound	7.04E+004	5.09E+004	5.24E+004	4.21E+005	6.67E+004	2.82E+004	1.13E−006	0.254
Upper bound	6.07E+005	5.12E+005	3.94E+005	3.94E+006	5.71E+005	4.96E+004	4.36E−006	0.685

*N1, effective population size of GNG, DPY, and SZZ; N2, effective population size of HTP; N3, effective population size of SZZ; NA, effective population size of ancestral population; t1, divergence time of N1 and N2 from ancestral population; t2, divergence time of N3 from N1 and N2; μ, mutation rate (per generation per locus)*.

### Ecological Niche Modeling

**Figure 4:**
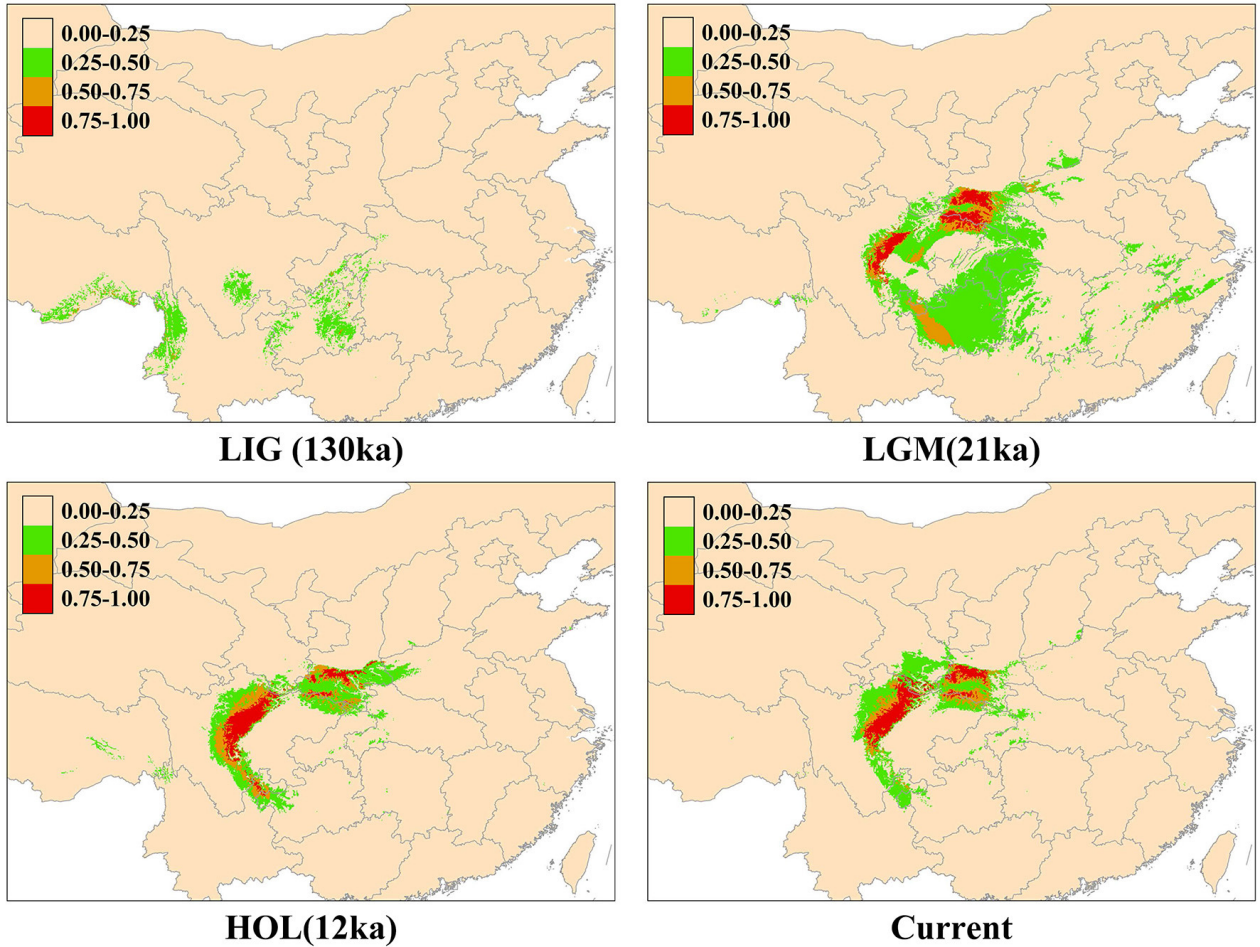
Predicted past and current distributions of *Rhinopithecus roxellana* based on ecological niche modeling using maxent: the current, the Holocene (HOL; 12 ka–current), the last glacial maximum (LGM; 18–21 ka), and the last interglacial period (LIG; 120–140 ka).

We obtained four distributions tendencies of the *Rhinopithecus roxellana*, including the LIG, LGM, HOL, and current models by maxent (Figure 4). All models had high predictive ability [area under the receiver operating characteristic curve (AUC) > 0.9]. The predicted model for the present potential range of *R. roxellana* was fairly congruent with the current distribution of the species. The ENMs suggested that the *R. roxellana* had population expansion during the LIG to LGM. Compared with the HOL and the current distributions, the current pattern had little diminished.

## Discussion

The present distributions of animal populations depend upon the historical processes and human activities. The habitat range of *Rhinopithecus roxellana* is a consequence of variation in ecological factors such as climatic change and intensity of human disturbance. In this study, we firstly used the interdisciplinary approaches to address the evolutionary dynamics of *R. roxellana* in Qinling Mountains. We examined genetic diversity, gene flow, genetic structures, and evolutionary history combination with the ENMs to elucidate ecological factors on the population demography.

### Genetic Diversity and Genetic Differentiation

We used microsatellite data to analyze the genetic diversity and population structure of *R. roxellana*. A relatively high level of genetic diversity had been found. Compared with previous studies based on the microsatellite profiles, the expected heterozygosity in all populations from our investigation (0.628) was close to that of the other studies of this species (0.559 in Li et al., 2020; 0.625 in Huang et al., 2016a; 0.591 in Chang et al., 2013; and 0.631 in Pan et al., 2005). In addition, there was no evidence of any past genetic bottlenecks in any of the sampled monkey bands (Table 2), revealing that these populations have not suffered significant population size reduction. This result was consistent with that of Li et al. (2020) and Huang et al. (2016a), which also focused on the monkeys in the Qinling Mountains. The *F*_ST_ and permutation test results revealed a genetic differentiation among different populations (*P* < 0.05) associated with topographical barriers such as mountain ridges, which might lead to the reduced gene flow between populations (Chang et al., 2013).

### Genetic Admixture and Gene Flow

The Bayesian clustering revealed three major subpopulations that strongly coincide with the major topographical ridge features in the study area. Our results indicated that the contemporary gene flow among the five populations was symmetric and low, whereas the historical gene flow for all pairs seemed asymmetric (Table 5). The possible explanations for the low contemporary gene flow were the more and more frequent human activities in this area. Human-constructed barriers such as rivers, villages, logging roads, and farmlands have limited the ability of BBs to freely move across these fragment landscapes for decades (Li et al., 2002; Wang et al., 2014). Moreover, the topography of the Qinling Mountains, which is dominated by high altitude temperate forest habitats, and open areas of fragmented and cleared forests containing villages and agricultural fields, may greatly increase the genetic differentiation of *R. roxellana* and restrict gene flow by decreasing the opportunities for successful dispersal (Wang et al., 2014). Nevertheless, satellite telemetry data indicated that some neighbor populations had small overlaps of their home ranges with occasional group fission–fusions (Qi et al., 2017). Meanwhile, the AMBs of *R. roxellana* played critical roles, which connected, gathered, and allocated gene flow among nearby populations (Li et al., 2020). But these only happened at a fine scale, which may explain the genetic migration of the bands between GNG, DPY, and SZZ (Figure 2C).

In general, gene flow is critical for mitigating the impacts of local adaptation by homogenizing populations from differing conditions or by spreading deleterious alleles across populations, and it also could spread favorable alleles to populations and increase genetic diversity (Welt et al., 2015; Epps and Keyghobadi, 2016). Previous research had reported that historical and contemporary gene migration was low among populations that often existed in highly fragmented habitats (Chiucchi and Gibbs, 2010), but if genetic structure was admixture, it might cause intense historical gene flow (Epps et al., 2013). However, whether the genetic structure of the Qinling Mountains was due to historical gene flow rather than contemporary ones needs further investigation.

### Demographic History

Our ABC demographic analysis detected three lineages (*N*1, *N*2, and *N*3) existing within the monkeys of the Qinling Mountains (*N*1: GNG, DPY, and SZZ; *N*2: CYG; and *N*3: HTP). The first divergence in our focal species that formed *N*1 and *N*3 lineages from ancestral linage was at 0.975 Ma [95% highest posterior density (HPD): 0.33–2.86 Ma]. Based on the genetic analysis and fossil data from existing populations, snub-nosed monkeys are distributed across eastern, central, southern, and southwestern China in the past 2 million years (Liedigk et al., 2003). In addition, our initial divergence time in the Qinling Mountains might occur at the Late Pleistocene. Due to climate change and orogenic movement, lots of numbers of animals have declined population sizes and even became extinct during this period (Buuveibaatar et al., 2016; Olsoy et al., 2016). Some species have been forced to shift their range to resist environmental changes and/or habit conversion (Ceballos, 2002; Cleland et al., 2012). The first divergence time estimation coincided with this historical period.

Moreover, the second divergence was at 0.23 Ma (95% HPD: 0.14–0.25 Ma), and formed *N*2, which originated from the second contact of the *N*1 and *N*3 lineages. Since the habitat fragmentation and ecological barriers could lead to restrict gene migration, some species would resist the threat of genetic homogeneity (Lenormand, 2002). It had been reported the monkeys would disperse long distance and build their families in another population (Huang et al., 2017). But so far, since our understanding of the mechanism against genetic homogeneity is still limited, further research would be needed. In addition, according to previous studies (Yu et al., 2016; Zhou et al., 2016; Kuang et al., 2019), one lineage of the Sichuan (SG) population was closely clustered with the Qinling (QL) population. This result indicated that the SG population may have contributed to the genetic structure of the QL population; for our subsequent research, this part would also be taken into account.

The ENM analyses also suggested that the golden snub-nosed monkeys had expanded their ranges during the LIG (0.14–0.12 Ma) to LGM. At the end of the largest glaciation (ca. 1.2–0.6 Ma), the temperature increased, and also, the relatively cold climate may have continued until the late Ionian stage (0.3–0.126 Ma) (Goldewijk et al., 2001). Thus, because the golden snub-nosed monkey could adapt to cold habitats (Yu et al., 2016), it is feasible that they expanded their ranges and increased their population sizes during this period.

It is necessary for us to raise the issues on a conservation strategy for the *R. roxellana*, in addition to carrying out further survey and studies on its ecology, behavior, and dietary selection. At the moment, what we know about the most possible threats could include road accidents (traffic is heavy in the areas), habitat loss, human's intentional killing due to economic reasons from the local culture, and natural disasters (Li et al., 2002; Wang et al., 2014). Although a series of natural reserve have been established in the Mt. Qinling, rapid tourism development would inevitably lead to the expanding of roads and other artificial constructions, while increasing human disturbance in this area. A monitoring system on their dynamic population profiles should be established, so that emergency strategies for their conservation could be applied if a significant population decline was detected. On the other hand, more technical programs for captive breeding are also required.

The Qinling Mountains are famous for their global biodiversity hotspot and refugia for many endangered mammals and birds, particularly during the last glaciation (Wang et al., 2014). The Chinese Government has invested a great deal of manpower to the conservation of “four precious species in the Mts. Qinling” (the giant panda, *Ailuropoda melanoleuca*; the golden snub-nosed monkey, *Rhinopithecus roxellana*; the crested ibis, *Nipponia nippon*; and the takin, *Budorcas taxicolor*). Our new results of the evolutionary history with the *R. roxellana* could also shed light on species with the same geographical distribution, as well as other threatened or endangered amphibians and reptiles in this area.

## Data Availability Statement

The datasets presented in this study can be found in online repositories. The names of the repository/repositories and accession number(s) can be found in the article/Supplementary Material.

## Ethics Statement

The animal study was reviewed and approved by Wildlife Protection Society of China.

## Author Contributions

BL and YL conceived the study. YL and KH performed the experiments. YL, LF, JY, and ZL contributed materials and analysis tools. BL, YL, and ST wrote the manuscript. LF and JY revised the manuscript. All authors approved the final version of the manuscript.

## Conflict of Interest

The authors declare that the research was conducted in the absence of any commercial or financial relationships that could be construed as a potential conflict of interest.

## Supplementary Material

Supplementary File 1 Genepop genotype file.

Supplementary Figure 1 The posterior probabilities of the 10 scenarios.

Supplementary Table 1 Microsatellite marker information.

Supplementary Table 2 Prior distributions for model parameters used in divergence model comparisons (Figure 3A).

Supplementary Table 3 The seven bioclimatic variables that were used in ecological niche modeling.
