# Supplementary material for: Genetic Structure and Evolutionary History of Rhinopithecus roxellana in Qinling Mountains, Central China
Source: Front Genet. 2021 Jan 20;11:611914. doi: 10.3389/fgene.2020.611914 (PMC7855588; doi:10.3389/fgene.2020.611914)
Supplement: Supplementary File 1 — Genepop genotype file. [file Table_1.DOCX]

G23' P9' P90' P26' P113' P7' P66' P103' P14' P15' P130' P40' P93' P25' P38' P142' P120'

H898 1 144 152 248 276 211 211 123 146 245 253 265 265 195 203 165 165 166 174 193 197 231 231 -9 -9 175 183 142 142 104 104 209 221 147 147

H932 1 144 148 276 276 211 211 123 123 249 253 265 265 195 195 165 169 166 166 185 193 231 231 281 285 -9 -9 138 142 104 104 221 221 147 147

H899 1 144 144 256 276 211 211 123 123 253 253 261 273 195 195 165 165 174 174 193 197 231 231 281 281 175 179 142 142 104 112 221 221 147 151

S1729 1 144 152 256 268 211 211 123 146 253 253 265 273 195 195 165 169 170 174 181 197 231 231 281 281 175 179 138 142 104 112 209 221 147 151

H947 1 144 152 256 276 211 211 123 127 245 257 249 265 195 195 165 169 166 174 185 185 231 231 281 281 175 175 142 142 104 104 209 213 147 147

H906 1 144 152 256 276 211 211 123 127 245 257 249 265 195 195 161 169 174 174 185 185 231 231 281 281 175 179 142 142 104 108 209 213 147 147

S1825 1 144 152 276 276 199 211 123 146 253 257 261 265 195 195 165 165 174 174 185 193 231 231 281 293 175 179 138 138 104 112 221 221 147 151

H916 1 152 156 256 276 211 211 123 127 245 257 249 265 195 195 165 169 166 174 185 197 231 231 281 293 179 179 138 142 104 104 209 221 147 159

S1813 1 152 152 256 268 211 211 123 123 253 257 249 265 195 203 165 169 166 174 193 197 231 243 281 293 179 179 142 142 104 104 209 221 151 159

H908 1 144 152 276 276 211 215 123 123 249 249 261 265 195 195 165 169 166 178 185 185 231 231 281 293 175 175 138 142 108 112 209 233 147 159

S1762 1 152 152 256 276 199 215 123 146 249 249 249 261 195 195 161 165 166 174 185 185 231 243 281 281 175 175 138 142 104 112 221 233 147 159

H910 1 144 152 256 276 211 215 123 123 253 257 265 269 195 195 165 165 162 166 185 197 231 243 281 281 175 175 142 142 112 112 209 209 147 147

S1757 1 152 152 256 268 211 211 123 146 253 257 249 265 195 195 161 165 166 174 185 185 231 243 281 285 175 175 142 142 104 112 209 221 147 151

H925 1 144 152 268 268 199 211 123 123 249 253 265 269 195 195 165 169 166 174 185 193 231 243 281 281 175 179 138 138 104 112 209 209 151 151

S1780 1 144 160 268 276 199 211 123 123 249 249 261 269 187 195 165 169 166 166 185 185 231 231 281 281 179 179 138 138 104 108 209 221 151 159

H918 1 152 152 268 276 211 211 123 123 249 253 269 269 195 203 165 169 166 174 185 185 231 231 293 293 179 179 138 142 104 112 209 209 147 159

S1763 1 144 152 268 276 211 211 123 123 253 253 269 269 195 203 165 169 166 174 185 193 231 231 281 293 175 179 138 138 104 104 -9 -9 147 151

H911 1 152 152 268 276 211 215 123 123 253 253 249 249 195 203 165 165 174 174 193 193 231 239 293 293 175 179 138 142 108 112 213 221 151 155

H931 1 152 152 256 268 211 211 123 123 253 257 249 261 195 195 165 165 174 174 185 193 231 239 293 293 179 179 138 142 108 108 221 233 147 155

S1707 1 152 152 276 276 211 215 123 123 245 253 249 273 195 203 165 169 162 174 193 197 223 231 281 281 175 179 142 142 104 112 -9 -9 147 147

S1708 1 152 152 268 276 211 211 123 123 245 253 269 277 195 203 161 169 162 174 185 193 223 243 281 293 175 179 138 142 104 112 -9 -9 155 159

H1060 1 152 152 276 276 211 215 123 146 245 253 249 269 195 203 169 169 162 174 185 193 231 231 281 293 179 183 142 142 104 104 209 221 147 155

S1756 1 148 152 276 276 211 211 123 146 245 245 249 265 195 195 161 169 174 174 185 185 231 231 293 293 175 179 142 142 104 108 221 233 151 155

S1847 1 144 152 252 276 211 211 123 123 245 253 261 269 187 203 161 165 166 174 185 193 -9 -9 -9 -9 179 183 138 138 104 104 -9 -9 -9 -9

S1739 1 144 156 256 276 211 211 123 123 245 245 249 269 195 195 161 165 166 174 193 193 231 231 281 293 179 179 138 138 104 112 209 209 147 147

S2330 1 152 152 -9 -9 199 211 123 123 249 253 261 265 -9 -9 165 169 166 166 185 185 -9 -9 281 285 179 179 138 142 104 108 -9 -9 147 147

S1782 1 152 156 268 276 199 211 123 123 245 249 265 269 195 203 161 165 162 166 185 193 231 231 281 293 175 179 138 138 104 104 221 221 147 151

S2397 1 144 152 256 276 199 199 123 146 253 253 265 265 195 195 161 169 166 166 185 197 231 231 281 281 179 179 142 142 104 104 -9 -9 151 151

S1767 1 152 152 268 276 199 199 -9 -9 245 249 265 269 195 203 165 169 174 174 185 197 231 231 293 293 179 179 134 138 108 112 209 233 151 151

H902 1 144 152 256 268 211 211 123 146 253 257 249 261 195 195 165 165 174 174 185 185 231 239 281 293 179 179 142 142 108 112 209 221 155 155

H909 1 144 152 268 276 211 215 123 123 253 253 249 261 195 195 161 165 174 174 193 193 231 239 293 293 175 179 138 142 108 108 209 221 147 155

H919 1 144 152 256 276 199 215 123 123 249 249 269 269 195 195 161 165 166 166 185 197 231 231 293 293 179 179 138 142 104 112 209 209 147 147

S1799 1 144 152 276 276 199 211 123 123 253 253 269 269 195 195 165 169 166 174 185 193 231 231 281 293 179 179 138 142 104 104 209 209 151 151

S1764 1 144 152 256 276 199 211 123 123 253 253 269 269 195 195 165 169 166 174 185 193 231 231 285 293 175 175 138 142 104 104 209 233 147 159

S1785 1 152 152 -9 -9 199 211 123 123 249 249 265 269 195 195 165 165 166 174 181 193 231 239 285 293 175 179 138 142 104 108 -9 -9 147 151

H927 1 144 144 256 268 199 211 123 123 257 257 261 269 195 195 161 169 174 174 185 185 239 243 281 281 175 179 138 138 104 104 209 221 151 151

H880 1 152 152 256 276 211 215 123 127 245 257 249 265 195 195 161 169 174 174 185 197 231 231 281 285 175 175 142 142 108 108 209 221 147 151

H876 1 148 152 256 276 199 211 123 127 245 257 265 265 195 203 169 169 166 174 185 185 231 231 281 281 175 175 138 138 108 112 221 221 151 151

S1787 1 152 152 276 276 211 215 123 127 245 257 249 249 195 203 161 169 174 174 197 197 231 231 285 293 175 175 142 142 104 108 221 221 147 151

S1790 1 144 148 248 276 211 211 123 146 249 257 261 265 195 195 165 169 174 174 193 197 231 231 281 281 179 183 138 142 104 112 209 221 147 147

H941 1 152 152 276 276 199 211 123 127 245 253 265 265 195 203 161 169 166 174 185 197 231 231 293 293 179 179 142 142 104 108 209 209 147 151

H905 1 144 152 268 276 211 215 119 146 253 253 265 265 195 203 165 173 166 166 181 193 231 231 281 285 175 175 138 142 104 104 221 221 151 155

H907 1 144 152 276 276 215 215 123 146 245 257 249 261 195 195 165 169 170 174 185 193 231 231 281 281 179 183 142 142 104 112 213 221 147 155

H928 1 152 152 268 276 211 215 123 123 245 253 261 265 195 203 165 169 166 174 185 193 231 231 281 285 175 175 138 138 104 108 213 221 147 151

H912 1 144 152 276 276 211 211 123 123 249 249 249 261 195 195 161 165 166 178 185 185 231 231 293 293 175 175 138 142 104 108 209 209 147 147

H978 1 144 152 256 276 211 211 123 123 253 257 261 261 195 195 165 169 166 174 185 193 231 243 281 293 175 175 138 138 104 112 221 221 151 159

H942 1 152 156 268 276 211 211 123 146 245 245 249 269 195 203 165 169 166 174 185 185 231 231 281 281 175 175 142 142 112 112 209 209 147 147

H913 1 144 152 276 276 211 211 123 123 253 253 269 269 195 203 169 169 166 174 193 193 231 231 281 293 175 179 138 138 104 108 209 209 147 151

S1858 1 152 152 248 268 199 199 123 146 249 253 249 265 203 203 161 169 166 166 193 193 243 243 281 293 175 179 138 142 104 112 209 221 147 147

H900 1 144 152 256 268 211 211 123 123 253 257 261 269 195 195 161 165 174 174 185 193 231 243 281 293 179 179 138 142 104 108 209 209 147 155

H893 1 152 152 248 276 199 211 123 123 249 253 269 269 203 203 169 169 162 166 185 193 231 243 281 293 179 179 138 138 104 104 209 209 147 151

H894 1 144 148 276 276 211 215 123 123 245 253 249 273 195 195 165 173 162 162 193 197 231 231 281 293 175 179 138 138 104 112 209 221 147 151

H939 1 148 152 276 276 199 211 123 127 245 257 249 265 195 195 161 169 174 174 185 197 231 231 281 293 175 179 138 142 108 108 209 221 147 151

S1887 1 144 152 252 276 199 211 123 123 253 253 261 265 195 195 161 165 166 174 185 197 231 231 281 293 175 179 134 138 108 112 -9 -9 151 151

H948 1 144 152 268 276 211 215 123 146 249 253 265 265 195 195 169 173 166 170 193 197 231 231 281 281 175 179 138 138 104 104 209 221 147 151

S1848 1 152 152 268 276 211 211 123 123 245 253 249 269 195 195 161 165 162 174 185 193 231 231 293 293 175 179 138 138 104 112 209 221 147 151

H979 1 152 156 252 276 211 215 123 123 245 249 265 269 195 195 165 165 166 170 185 185 231 231 281 293 175 179 138 138 104 104 209 209 147 151

H980 1 152 152 268 276 211 215 123 146 253 257 265 265 195 203 165 169 166 166 185 185 231 231 281 293 179 179 138 138 104 104 221 221 147 151

H920 1 144 148 248 276 199 211 123 146 249 257 249 261 195 195 169 169 174 174 197 197 231 243 281 285 175 175 138 142 108 112 221 229 147 147

H933 1 144 152 268 276 199 211 123 123 253 257 261 269 195 195 165 169 166 174 185 193 231 231 281 293 179 183 138 138 104 108 209 233 147 151

H949 1 144 152 256 268 211 211 123 123 249 249 261 269 195 195 165 165 166 174 193 193 231 243 285 293 175 179 138 142 104 112 209 233 147 147

H950 1 152 152 256 268 211 211 123 123 257 257 261 265 195 195 165 169 166 166 181 193 239 243 281 293 175 179 138 138 112 112 209 221 151 159

H951 1 144 152 256 268 199 215 123 127 245 253 249 265 195 195 161 161 174 174 193 197 231 243 281 293 175 179 142 142 108 112 209 221 147 151

H952 1 152 152 268 276 211 215 123 146 245 253 261 265 195 203 -9 -9 166 174 -9 -9 223 231 281 293 175 179 138 142 104 108 213 221 155 159

H953 1 152 160 268 276 211 211 123 123 253 253 261 269 187 195 169 169 162 166 185 193 231 231 281 281 179 179 138 142 104 108 209 221 147 159

H954 1 152 156 256 276 211 211 123 123 245 257 249 265 195 203 165 169 166 174 -9 -9 231 231 293 293 175 179 138 142 104 104 209 221 147 151

H955 1 152 152 268 276 211 211 123 123 253 253 261 265 203 203 161 169 166 174 185 185 231 243 281 281 175 179 138 142 104 108 209 221 147 147

H956 1 152 152 256 276 211 211 123 123 245 253 249 273 195 195 -9 -9 166 166 -9 -9 231 231 281 281 175 179 138 138 104 104 209 213 155 159

H957 1 152 152 248 276 211 211 123 127 245 249 249 265 195 195 165 169 166 174 -9 -9 231 231 281 293 175 179 138 142 104 104 209 221 147 147

H958 1 152 152 276 276 211 211 123 123 245 249 265 273 195 195 161 165 166 166 185 185 231 231 281 293 175 175 138 142 104 112 213 221 155 159

H959 1 152 152 256 276 211 211 123 123 245 253 249 273 195 195 -9 -9 166 166 -9 -9 231 231 281 281 175 179 138 138 104 104 209 213 151 159

H960 1 144 152 252 256 199 211 123 127 249 257 249 249 195 195 169 169 166 166 -9 -9 231 231 281 293 175 179 138 142 104 104 209 209 147 151

H961 1 152 152 256 256 211 211 123 146 253 257 261 265 195 195 165 165 166 166 181 185 231 243 293 293 175 175 138 138 104 104 221 233 151 151

H962 1 152 152 248 276 211 211 123 127 245 249 249 265 195 195 165 165 166 174 -9 -9 231 231 281 293 175 179 138 142 104 104 -9 -9 147 147

H963 1 152 152 252 276 215 215 123 123 249 253 265 269 195 195 161 165 166 174 -9 -9 231 231 -9 -9 175 179 138 138 104 104 221 233 151 151

H964 1 152 152 248 276 211 211 123 127 245 249 249 265 195 195 165 169 166 174 -9 -9 231 231 281 293 175 179 138 142 104 104 209 221 147 147

S2001 1 144 156 256 276 211 211 123 127 245 257 265 269 195 195 165 165 166 166 185 185 231 231 281 281 175 179 138 142 104 112 209 213 147 151

H1045 1 152 152 268 276 211 211 123 146 245 253 265 265 195 203 165 169 166 170 185 185 231 231 293 293 175 179 138 138 104 104 221 221 151 159

S1987 1 144 148 244 248 211 211 123 123 249 253 265 265 195 195 165 173 166 170 181 197 231 231 281 281 179 179 138 138 -9 -9 -9 -9 147 151

S2015 1 -9 -9 -9 -9 211 211 123 146 253 257 -9 -9 195 195 165 165 166 170 -9 -9 231 231 293 293 175 179 138 138 104 104 -9 -9 147 151

S2005 1 144 152 268 268 211 215 123 146 253 253 249 265 195 203 165 173 166 174 185 193 231 243 281 285 175 179 138 142 104 108 -9 -9 147 151

S2039 1 -9 -9 -9 -9 211 211 123 146 245 253 249 265 195 203 161 165 166 174 185 185 231 231 281 293 175 179 142 142 112 112 -9 -9 147 159

S2026 1 152 152 268 276 211 211 -9 -9 253 253 261 265 -9 -9 161 165 -9 -9 185 193 -9 -9 281 293 175 179 138 142 104 112 -9 -9 147 151

H1003 1 152 152 256 256 211 211 123 127 253 257 261 265 195 195 165 165 166 166 181 185 231 243 293 293 175 175 138 138 104 104 221 233 151 151

S2834 2 -9 -9 -9 -9 211 211 -9 -9 249 249 265 265 -9 -9 161 169 -9 -9 -9 -9 -9 -9 277 277 -9 -9 138 138 -9 -9 -9 -9 143 147

S2835 2 152 152 -9 -9 211 211 123 123 245 249 265 265 -9 -9 161 169 166 166 -9 -9 213 217 277 285 175 179 138 142 -9 -9 -9 -9 147 151

S2839 2 -9 -9 268 268 211 211 -9 -9 249 249 261 265 -9 -9 -9 -9 166 170 -9 -9 -9 -9 273 277 -9 -9 138 138 -9 -9 -9 -9 147 151

S2840 2 148 152 268 276 199 211 127 127 249 261 269 269 -9 -9 161 169 166 170 197 197 -9 -9 285 293 -9 -9 134 138 -9 -9 -9 -9 147 151

S2841 2 152 152 276 276 199 211 123 127 249 249 261 265 -9 -9 161 161 166 166 181 185 231 231 281 293 175 179 134 142 -9 -9 -9 -9 147 147

S2842 2 152 152 276 276 199 211 123 127 245 249 261 265 -9 -9 161 161 166 166 181 185 231 231 281 293 175 179 134 142 100 100 -9 -9 147 147

S2843 2 152 152 288 288 211 211 123 146 249 257 261 269 195 195 169 173 166 170 -9 -9 231 231 289 293 175 183 138 142 -9 -9 -9 -9 147 151

S2844 2 160 160 268 288 199 211 -9 -9 -9 -9 261 269 -9 -9 169 177 -9 -9 -9 -9 -9 -9 273 277 -9 -9 138 142 100 104 209 229 151 151

S2845 2 156 156 268 288 211 211 -9 -9 -9 -9 261 265 -9 -9 169 177 158 170 185 197 217 217 273 277 167 167 138 146 -9 -9 209 229 147 147

S2847 2 152 152 268 268 -9 -9 -9 -9 249 249 269 269 195 203 169 177 166 170 197 197 217 217 277 277 175 175 138 146 104 104 209 233 147 151

S2848 2 152 152 -9 -9 199 211 -9 -9 245 249 261 265 -9 -9 169 177 166 170 185 193 231 231 277 277 175 179 138 138 -9 -9 -9 -9 147 147

S2849 2 -9 -9 268 288 -9 -9 -9 -9 249 249 261 265 -9 -9 161 169 158 170 -9 -9 -9 -9 277 277 -9 -9 138 146 -9 -9 229 229 -9 -9

S2851 2 148 160 288 288 -9 -9 -9 -9 245 249 261 261 -9 -9 169 177 166 166 -9 -9 -9 -9 273 277 -9 -9 138 138 100 100 209 229 151 151

S2852 2 -9 -9 260 260 199 215 123 123 257 257 261 265 203 203 161 169 166 166 185 197 231 231 281 293 175 179 138 142 100 112 209 229 147 151

S2855 2 148 160 -9 -9 199 211 123 127 249 249 261 265 195 195 169 169 166 166 185 185 231 231 277 277 -9 -9 138 146 100 104 -9 -9 147 147

S2856 2 -9 -9 268 288 207 211 -9 -9 -9 -9 261 265 -9 -9 161 177 166 166 -9 -9 231 239 277 277 -9 -9 138 146 -9 -9 -9 -9 147 151

S2858 2 -9 -9 288 288 203 211 -9 -9 245 245 261 265 -9 -9 161 169 166 170 197 197 -9 -9 277 277 -9 -9 138 146 100 104 -9 -9 147 159

S2860 2 156 156 268 276 -9 -9 123 127 245 245 261 265 203 203 -9 -9 166 174 -9 -9 231 231 277 277 175 179 138 142 -9 -9 209 209 147 147

S2861 2 -9 -9 256 288 211 211 123 123 249 249 261 265 195 203 173 173 166 178 185 197 231 231 277 289 175 183 134 142 104 112 209 229 147 147

S2862 2 152 152 268 276 199 199 123 127 245 245 261 265 203 203 165 165 166 174 185 185 231 231 281 285 175 179 134 142 104 112 181 213 147 147

S2863 2 152 152 260 276 215 215 123 123 245 257 261 261 -9 -9 161 169 166 174 -9 -9 231 243 -9 -9 167 167 138 146 -9 -9 -9 -9 147 147

S2864 2 152 152 252 288 199 211 123 123 249 261 261 269 203 203 161 169 166 170 197 197 217 231 277 277 175 183 138 146 -9 -9 209 209 151 151

S2865 2 152 160 -9 -9 199 211 123 127 245 245 265 265 -9 -9 169 177 166 166 185 185 231 231 277 277 179 183 138 146 -9 -9 209 209 147 147

S2866 2 -9 -9 276 288 211 219 -9 -9 -9 -9 261 265 -9 -9 169 169 166 170 185 197 -9 -9 277 277 175 175 138 138 -9 -9 209 209 147 147

S2867 2 152 168 260 276 199 211 123 146 245 257 265 265 -9 -9 169 169 166 166 185 197 231 231 277 277 -9 -9 138 146 104 112 209 229 147 147

S2868 2 152 152 268 268 207 211 123 127 257 261 261 265 -9 -9 169 177 166 166 197 197 231 231 273 277 175 175 138 142 -9 -9 209 229 147 151

S2869 2 152 160 276 276 -9 -9 -9 -9 249 257 261 261 -9 -9 161 169 166 174 -9 -9 -9 -9 277 289 179 183 138 142 -9 -9 209 209 147 147

S2873 2 152 160 260 288 211 211 123 123 249 249 265 265 187 203 165 169 166 166 185 185 223 231 285 293 175 183 138 142 104 112 229 233 151 151

S2874 2 148 152 252 276 211 211 123 127 249 261 261 269 195 203 -9 -9 166 170 181 181 223 231 285 293 -9 -9 134 142 104 112 -9 -9 -9 -9

S2875 2 152 152 256 276 211 211 123 123 249 249 261 261 195 203 165 165 166 174 185 189 231 243 289 293 183 183 -9 -9 104 112 181 205 151 155

S2876 2 152 152 260 276 211 211 123 127 245 257 265 265 195 203 165 165 166 166 181 189 231 231 281 289 175 179 138 138 104 104 221 221 151 155

S2877 2 152 152 252 276 211 211 123 123 245 261 261 265 195 195 165 169 166 170 181 197 231 243 289 293 175 179 142 142 104 112 225 225 151 155

S2879 2 152 152 252 260 211 211 123 123 249 249 265 265 191 191 165 169 166 166 185 189 231 243 285 293 175 183 134 142 104 112 225 225 155 155

S2880 2 152 160 260 276 211 211 123 123 249 257 261 261 187 203 165 169 166 166 181 189 223 231 293 293 175 175 138 142 104 112 205 221 155 155

S2881 2 152 152 252 276 -9 -9 123 123 245 249 265 265 195 203 165 165 166 166 -9 -9 223 231 285 293 175 179 138 138 104 104 205 217 151 155

S2882 2 152 152 260 276 211 211 123 123 245 249 261 265 195 203 169 169 166 166 -9 -9 231 231 293 293 175 179 138 142 104 104 181 221 147 151

S2883 2 152 152 252 276 199 211 123 123 245 249 261 265 -9 -9 169 169 166 166 197 197 231 231 281 293 175 179 142 142 104 104 181 181 155 155

S2884 2 152 160 260 260 211 211 123 127 249 249 261 265 187 195 165 169 166 166 185 185 -9 -9 281 293 175 175 138 138 104 112 205 213 155 155

S2885 2 156 156 260 260 199 211 127 139 249 249 265 269 195 203 165 169 166 166 185 193 -9 -9 293 293 175 175 142 142 104 104 205 213 151 155

S2886 2 152 152 276 288 211 211 123 127 245 249 261 265 203 203 165 165 166 174 -9 -9 -9 -9 281 293 175 179 -9 -9 104 112 181 181 151 151

S2887 2 152 152 260 276 199 215 123 123 245 257 261 261 195 203 169 169 166 174 -9 -9 231 243 293 293 175 179 142 142 108 112 -9 -9 151 151

S2888 2 148 152 260 260 211 215 123 123 257 257 261 265 199 203 169 169 166 166 185 185 231 231 281 293 175 179 142 142 104 112 213 213 151 155

S2889 2 152 152 -9 -9 -9 -9 123 146 245 249 261 261 195 203 169 169 166 166 -9 -9 -9 -9 285 293 175 179 138 142 104 104 205 225 151 155

S2890 2 152 152 252 260 199 215 123 139 249 249 261 265 195 203 165 169 166 174 181 197 -9 -9 -9 -9 175 183 138 138 104 104 209 209 151 151

S2891 2 148 152 260 276 199 215 123 123 249 257 261 261 -9 -9 165 169 166 174 -9 -9 -9 -9 281 293 179 183 138 142 104 112 221 221 151 155

S2892 2 152 160 252 260 211 211 123 123 249 257 261 261 -9 -9 165 169 174 174 185 197 223 223 293 293 175 183 138 138 104 112 205 217 151 155

S2893 2 152 152 260 276 199 211 123 146 245 257 265 265 203 203 165 169 166 166 185 185 231 231 281 293 175 175 138 138 104 112 -9 -9 151 151

S2896 2 152 152 260 276 -9 -9 123 123 249 249 261 265 203 203 169 169 166 166 -9 -9 223 231 293 293 175 175 142 142 104 112 209 221 151 151

S2897 2 152 152 260 276 199 215 123 123 245 249 265 265 183 187 165 169 166 170 197 197 223 231 293 293 175 179 142 142 -9 -9 205 213 151 155

S2898 2 -9 -9 260 276 199 211 123 127 245 257 261 261 183 187 161 169 166 170 181 185 231 243 293 293 175 179 -9 -9 104 108 205 205 151 151

S2899 2 152 152 252 276 215 215 123 127 249 261 261 269 183 187 165 169 166 170 197 197 231 243 277 293 179 179 142 142 104 104 181 213 155 155

S2900 2 -9 -9 256 260 199 215 123 123 245 249 261 261 187 199 165 169 166 170 185 197 -9 -9 293 293 179 183 142 142 104 112 181 205 -9 -9

S2904 2 156 156 260 260 199 211 127 139 249 249 265 269 191 191 165 169 166 166 185 197 231 243 293 293 175 175 138 142 104 104 205 217 151 155

S2905 2 -9 -9 256 276 199 211 123 127 -9 -9 -9 -9 191 191 165 165 166 170 185 197 -9 -9 281 289 175 183 -9 -9 104 104 -9 -9 -9 -9

S2906 2 148 152 276 276 211 215 123 123 245 249 261 261 195 203 165 165 166 166 185 197 -9 -9 285 293 179 183 134 134 -9 -9 205 205 151 155

S2908 2 -9 -9 260 276 199 199 123 123 245 257 261 265 195 203 165 169 166 170 185 185 223 231 285 293 175 179 134 142 104 112 221 221 151 155

S2909 2 152 152 260 276 215 215 123 123 249 257 261 265 195 203 165 165 166 170 181 197 223 231 285 293 175 175 142 142 104 104 213 213 151 155

S2912 2 152 152 276 276 -9 -9 123 127 249 249 265 265 191 203 165 165 166 166 -9 -9 231 231 281 293 175 179 134 142 104 112 181 221 151 151

S2913 2 -9 -9 268 268 -9 -9 123 127 245 261 265 265 -9 -9 165 169 166 170 -9 -9 231 243 293 293 175 179 142 142 104 104 181 225 151 155

S2915 2 -9 -9 276 276 215 219 123 139 245 245 265 265 195 203 165 169 170 174 189 197 231 231 281 281 183 183 138 138 104 104 -9 -9 151 151

S2917 2 152 152 248 276 211 219 123 127 245 245 265 265 195 203 165 165 166 166 197 197 231 231 281 289 179 183 142 142 104 104 -9 -9 151 151

S2918 2 144 152 256 276 199 211 123 123 245 261 265 273 187 195 165 169 166 170 -9 -9 -9 -9 289 293 175 179 142 142 104 104 -9 -9 -9 -9

S2919 2 152 152 252 276 211 219 123 123 249 249 261 265 -9 -9 165 169 166 174 -9 -9 223 231 289 293 175 175 138 138 104 112 -9 -9 151 151

S2921 2 156 156 260 276 199 215 123 123 245 249 265 265 203 203 165 169 166 174 197 197 223 231 281 289 175 175 138 138 104 112 205 221 151 155

S2922 2 -9 -9 276 276 211 219 123 127 245 257 261 261 195 203 165 165 166 174 -9 -9 231 231 281 289 175 183 138 142 104 112 -9 -9 151 155

S2923 2 -9 -9 260 260 199 211 123 127 249 257 261 265 195 203 165 165 166 174 185 185 -9 -9 289 293 175 179 138 138 -9 -9 181 181 151 155

S2926 2 152 152 -9 -9 199 211 123 123 249 261 261 261 203 203 169 169 166 174 193 197 231 243 -9 -9 167 179 138 142 104 112 -9 -9 151 151

S2927 2 152 168 252 276 211 211 123 123 249 261 261 269 -9 -9 169 169 166 174 185 197 243 243 289 293 179 179 138 142 104 112 -9 -9 151 151

S2928 2 152 152 276 288 199 211 123 127 245 257 261 261 195 203 165 177 166 174 -9 -9 231 231 281 289 175 183 138 142 112 112 -9 -9 147 151

S2929 2 -9 -9 -9 -9 211 215 123 142 249 257 261 265 -9 -9 169 177 166 170 185 185 -9 -9 -9 -9 175 179 138 146 -9 -9 -9 -9 151 151

S2930 2 152 160 260 260 211 211 123 123 249 249 261 265 187 203 165 169 166 166 185 185 223 231 281 293 183 183 138 138 104 104 -9 -9 155 155

S2645 3 -9 -9 252 276 199 215 123 123 253 253 261 261 -9 -9 165 165 162 166 185 197 -9 -9 281 293 179 183 134 142 -9 -9 -9 -9 -9 -9

S2654 3 -9 -9 276 284 211 215 123 127 249 249 261 269 -9 -9 165 165 166 166 185 185 223 235 277 281 175 175 138 138 -9 -9 205 213 147 151

S2655 3 152 160 240 292 199 215 123 123 253 257 261 261 187 195 161 165 166 166 185 185 231 235 277 281 179 187 138 142 112 112 205 213 147 151

S2656 3 152 160 252 260 211 219 123 139 249 249 261 265 195 195 165 165 166 166 197 197 223 235 281 285 175 175 138 142 -9 -9 205 213 147 147

S2657 3 152 168 274 292 199 199 123 123 245 253 261 261 195 195 165 169 158 166 185 197 231 231 281 289 175 179 134 142 112 112 205 205 147 147

S2658 3 152 160 292 296 215 219 123 139 -9 -9 261 265 -9 -9 165 169 166 166 185 197 223 235 277 285 175 175 138 142 -9 -9 205 205 147 171

S2659 3 -9 -9 274 280 211 215 127 127 249 249 261 269 -9 -9 165 165 166 166 185 197 235 235 273 289 175 175 138 138 -9 -9 205 205 -9 -9

S2660 3 152 160 268 276 211 219 123 123 249 253 261 261 187 187 161 165 166 170 181 197 231 243 277 293 179 183 134 142 100 100 205 213 147 151

S2661 3 144 152 256 276 211 215 123 139 249 253 265 265 195 195 165 169 166 166 185 197 231 235 277 281 175 175 138 142 100 100 -9 -9 147 147

S2662 3 152 160 256 276 211 215 123 131 245 257 261 265 187 203 165 169 166 166 185 185 223 223 293 293 175 179 138 138 108 108 -9 -9 151 159

S2664 3 -9 -9 292 292 203 203 131 131 -9 -9 261 261 191 199 161 165 166 170 181 185 -9 -9 277 289 175 175 138 138 -9 -9 205 213 -9 -9

S2665 3 152 160 260 292 199 215 123 131 253 257 261 261 187 195 165 169 166 166 185 185 235 239 281 293 179 187 142 142 104 108 205 209 147 151

S2666 3 152 156 260 276 211 219 123 139 253 257 261 261 187 195 169 169 166 166 185 197 223 231 281 281 175 183 134 138 -9 -9 -9 -9 147 155

S2668 3 152 160 252 276 211 215 123 139 245 257 265 265 187 187 165 169 166 166 185 197 223 231 281 281 175 175 142 142 104 108 205 213 151 155

S2669 3 152 160 276 276 199 211 123 123 245 253 261 261 187 195 165 169 166 166 185 193 231 239 277 281 175 187 134 142 112 112 -9 -9 147 151

S2670 3 152 160 276 276 215 219 123 123 249 249 261 269 191 199 165 165 166 166 185 197 223 235 281 281 179 179 142 142 -9 -9 -9 -9 147 151

S2671 3 152 160 256 276 215 219 123 123 249 257 261 261 187 191 165 169 162 166 185 197 223 231 281 281 175 175 138 138 104 108 205 205 151 159

S2673 3 152 160 256 260 199 199 123 123 257 257 265 265 187 195 165 165 166 174 185 197 231 235 281 281 179 183 138 142 112 112 -9 -9 147 151

S2674 3 152 152 252 268 199 215 -9 -9 253 257 -9 -9 -9 -9 -9 -9 166 170 -9 -9 -9 -9 277 281 175 175 138 138 -9 -9 -9 -9 151 151

S2676 3 152 160 256 276 199 199 123 123 253 261 261 265 187 195 165 165 166 174 185 197 231 239 281 293 179 187 138 142 104 112 -9 -9 151 151

S2678 3 -9 -9 -9 -9 211 211 -9 -9 249 257 -9 -9 -9 -9 165 165 170 174 -9 -9 -9 -9 -9 -9 179 179 138 142 -9 -9 -9 -9 -9 -9

S2681 3 148 152 248 276 215 219 123 123 253 253 -9 -9 -9 -9 -9 -9 162 166 -9 -9 -9 -9 281 285 175 179 142 142 -9 -9 -9 -9 151 151

S2682 3 152 156 260 276 211 219 -9 -9 -9 -9 261 261 -9 -9 165 165 166 170 -9 -9 -9 -9 281 285 175 183 134 142 -9 -9 -9 -9 147 155

S2683 3 152 152 260 276 211 211 -9 -9 253 257 -9 -9 -9 -9 165 165 162 166 -9 -9 -9 -9 277 285 175 175 138 142 -9 -9 221 221 151 155

S2686 3 152 152 256 260 211 219 123 131 249 257 -9 -9 187 203 165 165 166 166 -9 -9 -9 -9 293 293 175 175 142 142 104 108 -9 -9 -9 -9

S2687 3 152 152 252 252 211 215 123 123 249 257 261 261 195 195 165 165 162 166 197 197 223 231 281 281 179 183 138 142 104 108 -9 -9 151 151

S2690 3 152 160 260 276 211 215 123 139 253 257 261 261 187 195 165 165 166 166 185 197 231 231 281 293 175 187 142 142 104 108 -9 -9 151 151

S2691 3 152 152 252 256 211 215 123 131 249 257 -9 -9 187 195 165 165 162 166 185 197 -9 -9 281 293 175 179 142 142 104 108 -9 -9 151 155

S2692 3 144 152 260 276 211 215 123 139 253 253 -9 -9 195 195 165 169 166 166 185 197 -9 -9 281 281 175 175 142 142 104 104 -9 -9 -9 -9

S2695 3 152 160 260 268 199 211 123 123 249 257 261 261 187 195 165 169 166 166 197 197 231 243 281 281 175 183 134 142 108 108 -9 -9 147 155

S2697 3 152 156 252 276 211 219 123 123 253 257 261 269 187 203 161 165 166 166 197 197 -9 -9 281 293 -9 -9 138 142 104 108 -9 -9 -9 -9

S2699 3 152 156 252 276 199 211 123 123 253 257 -9 -9 187 195 165 165 166 166 185 197 -9 -9 281 293 -9 -9 142 142 108 112 -9 -9 147 151

S2701 3 152 156 260 276 215 219 139 139 249 253 -9 -9 187 195 165 169 166 166 185 197 223 239 281 285 175 175 142 142 104 104 205 209 147 147

S2742 4 148 152 -9 -9 207 211 123 123 249 253 261 261 191 199 161 169 166 170 189 193 239 243 281 289 175 179 134 134 -9 -9 -9 -9 147 151

S2744 4 140 160 260 260 207 227 -9 -9 257 257 261 273 195 195 165 169 162 166 181 189 223 227 277 277 175 175 138 138 100 104 205 205 155 159

S2745 4 152 156 292 292 207 215 131 146 253 257 257 273 -9 -9 161 169 158 166 197 197 223 227 277 281 183 183 138 138 -9 -9 205 213 143 155

S2746 4 152 152 -9 -9 215 227 131 139 249 265 261 273 195 199 161 165 166 170 185 193 223 243 277 281 167 175 138 142 -9 -9 -9 -9 155 159

S2747 4 152 152 -9 -9 203 227 131 135 249 257 265 273 187 191 161 165 158 166 193 193 223 227 273 277 175 183 138 138 104 104 205 205 159 159

S2748 4 152 152 240 264 -9 -9 131 139 249 257 273 273 187 195 169 169 162 166 189 189 223 243 285 289 167 167 134 138 100 104 205 213 143 147

S2749 4 152 152 -9 -9 211 215 123 127 265 265 261 265 183 187 161 161 158 170 197 197 235 235 -9 -9 167 175 138 138 104 104 205 205 155 159

S2750 4 148 148 240 256 211 227 127 131 249 265 261 273 187 199 161 161 158 170 185 193 235 235 277 289 167 167 138 138 108 108 205 205 143 155

S2751 4 156 156 240 256 211 215 131 131 249 257 269 273 187 199 161 165 158 170 193 193 223 239 273 277 167 167 138 138 100 100 -9 -9 155 159

S2752 4 152 152 -9 -9 211 215 123 131 253 257 265 265 199 199 161 161 162 166 189 189 223 235 277 277 167 179 138 138 108 108 205 205 159 159

S2754 4 152 152 240 240 215 215 123 131 249 257 265 269 187 199 165 169 158 166 189 193 235 235 281 281 175 183 -9 -9 104 108 205 213 143 147

S2755 4 152 168 -9 -9 211 227 123 131 249 249 261 265 187 199 161 169 162 166 -9 -9 223 235 277 289 167 175 -9 -9 112 112 213 213 171 171

S2756 4 148 152 288 288 215 227 127 131 249 265 261 265 187 199 161 165 158 170 197 197 223 231 277 277 167 175 138 138 108 108 -9 -9 171 171

S2757 4 148 156 292 292 211 215 131 131 265 265 265 273 187 187 161 165 166 170 185 193 223 243 277 277 167 175 138 138 108 108 213 213 -9 -9

S2758 4 152 152 -9 -9 215 227 131 146 253 257 273 277 187 187 169 169 158 170 193 193 223 243 277 277 167 167 138 138 104 108 205 205 155 171

S2759 4 152 152 -9 -9 215 227 131 131 249 257 273 277 183 187 161 165 166 166 189 189 223 243 -9 -9 167 175 138 138 112 112 213 213 171 171

S2760 4 152 152 292 292 203 227 123 135 253 257 265 277 183 187 161 161 166 170 -9 -9 223 239 277 281 167 179 -9 -9 108 108 -9 -9 151 171

S2761 4 148 148 -9 -9 215 215 123 131 249 253 265 269 187 199 165 169 158 170 189 193 223 239 277 289 175 183 134 138 108 108 -9 -9 147 171

S2762 4 152 160 -9 -9 215 215 123 135 253 257 261 273 187 187 165 165 166 166 181 189 -9 -9 277 289 167 183 138 138 112 112 205 213 171 171

S2763 4 152 168 292 292 207 211 123 127 249 249 261 277 183 199 161 169 -9 -9 189 189 223 235 277 277 167 175 138 138 104 112 205 205 171 171

S2764 4 148 152 264 292 207 215 131 146 257 257 277 277 187 199 161 161 166 170 189 193 -9 -9 273 277 175 183 138 138 104 108 205 205 143 159

S2765 4 148 152 240 292 211 227 123 127 249 257 265 277 183 187 161 165 166 170 189 189 223 231 -9 -9 167 175 138 138 104 108 205 205 -9 -9

S2766 4 144 152 -9 -9 215 227 123 135 257 257 261 277 183 199 169 169 166 170 193 193 223 243 273 277 175 179 138 138 104 112 205 205 143 171

S2767 4 152 152 -9 -9 203 215 123 127 249 249 265 277 199 199 161 165 166 170 189 193 223 231 273 277 179 179 138 138 104 108 -9 -9 155 155

S2768 4 148 148 256 260 207 211 131 131 249 257 261 277 187 187 165 169 166 170 189 189 223 231 273 277 175 179 134 138 104 112 -9 -9 147 159

S2769 4 152 152 244 292 211 215 127 139 249 257 265 273 187 187 165 165 166 170 -9 -9 223 243 277 277 167 175 134 138 100 100 -9 -9 147 147

S2770 4 148 160 276 292 215 227 123 131 249 261 -9 -9 -9 -9 161 165 158 170 193 193 223 243 277 277 167 167 138 138 108 108 205 205 171 171

S2771 4 148 160 260 292 215 227 131 139 -9 -9 265 277 183 199 161 169 158 170 189 193 223 243 277 277 167 179 138 138 104 108 -9 -9 147 155

S2772 4 148 152 264 292 215 227 123 123 253 253 261 265 199 199 -9 -9 158 166 181 189 223 243 273 277 167 175 138 138 112 112 205 205 147 159

S2773 4 152 160 292 292 215 215 123 127 257 257 261 265 187 187 161 165 158 166 181 189 223 223 273 277 175 179 138 138 108 108 205 205 147 155

S2775 4 148 152 -9 -9 215 215 131 139 257 261 261 269 183 187 161 165 166 170 189 189 223 223 281 281 175 183 134 134 108 108 -9 -9 -9 -9

S2784 4 148 148 248 260 215 215 123 123 249 257 269 269 187 199 165 169 158 170 189 193 223 231 281 281 167 167 134 134 -9 -9 -9 -9 147 147

S2787 4 156 156 -9 -9 211 215 127 131 -9 -9 261 269 187 199 -9 -9 162 166 -9 -9 223 231 281 281 167 179 -9 -9 -9 -9 -9 -9 -9 -9

S2789 4 148 156 264 288 215 215 123 127 253 257 269 277 187 199 161 165 166 166 189 189 223 243 272 281 175 183 134 138 112 112 205 213 147 155

S2790 4 148 148 248 260 215 215 123 123 249 257 265 269 187 199 169 169 158 170 189 193 223 243 272 281 179 179 134 134 108 108 -9 -9 171 171

S2792 4 152 152 256 268 211 215 123 123 257 257 265 273 187 199 161 161 166 166 193 193 223 243 272 281 175 183 134 138 104 112 -9 -9 147 159

S2796 4 148 152 252 256 211 215 123 123 257 257 261 261 187 187 161 165 166 166 181 189 223 223 281 289 175 183 134 134 112 112 -9 -9 147 151

S2798 4 144 148 252 260 207 215 123 131 253 257 261 269 187 199 161 165 162 166 181 189 223 223 281 281 175 183 134 134 104 108 205 213 147 151

S2800 4 148 152 248 264 207 215 135 139 257 257 261 261 187 199 161 165 166 166 181 189 223 231 281 281 167 175 134 134 104 112 205 213 147 147

S2801 4 148 156 -9 -9 211 215 -9 -9 257 257 269 269 187 199 -9 -9 166 170 189 193 223 243 272 281 175 183 134 134 108 108 -9 -9 147 147

S2802 4 152 152 260 264 211 215 123 131 253 257 261 269 199 199 165 169 166 166 189 189 223 227 -9 -9 175 175 134 134 108 108 -9 -9 155 159

S2804 4 148 160 -9 -9 -9 -9 123 131 253 257 273 273 -9 -9 -9 -9 166 166 185 189 223 243 272 277 175 183 138 138 104 112 -9 -9 155 159

S2806 4 144 148 248 260 211 215 127 139 249 257 261 273 187 195 161 165 166 170 185 185 223 227 281 285 167 175 134 134 104 104 -9 -9 147 147

S2808 4 148 152 -9 -9 211 215 -9 -9 253 261 273 277 199 199 161 165 162 166 -9 -9 -9 -9 -9 -9 -9 -9 134 138 -9 -9 -9 -9 147 147

S2810 4 148 152 252 252 211 215 127 131 253 261 261 269 187 199 165 165 162 166 189 189 223 227 281 281 175 175 134 138 -9 -9 -9 -9 147 147

S2811 4 148 148 256 260 215 215 131 135 253 257 261 269 199 199 161 161 166 166 185 189 223 243 281 281 175 183 134 134 108 108 -9 -9 155 159

S2812 4 152 152 -9 -9 215 215 139 139 249 257 261 265 187 195 161 165 166 166 185 193 223 243 281 281 175 179 134 134 104 104 -9 -9 155 159

S2814 4 -9 -9 256 260 211 215 123 127 257 257 261 265 187 199 161 165 166 170 189 189 223 243 281 289 175 175 138 138 104 104 -9 -9 155 159

S2815 4 148 152 252 260 207 215 123 131 253 257 261 269 187 199 165 165 162 166 185 189 223 223 281 281 167 167 134 134 104 108 -9 -9 143 147

S2817 4 148 152 252 256 -9 -9 -9 -9 249 257 261 261 187 195 161 161 -9 -9 -9 -9 -9 -9 272 272 -9 -9 -9 -9 104 104 -9 -9 -9 -9

S2818 4 148 160 252 260 211 215 127 139 249 253 261 261 199 199 161 165 166 170 185 189 223 227 281 285 175 175 134 134 108 108 -9 -9 143 147

S2820 4 148 160 248 252 211 215 123 135 257 257 273 277 199 199 161 169 166 170 189 189 -9 -9 281 281 175 175 134 134 112 112 -9 -9 155 159

S2822 4 148 152 256 260 215 215 123 139 249 257 265 265 187 195 165 165 166 166 189 193 -9 -9 281 281 167 175 134 134 104 104 -9 -9 155 159

S2823 4 148 148 256 260 207 211 123 123 249 257 261 261 187 199 161 169 166 170 189 189 223 243 272 272 175 179 134 134 112 112 -9 -9 147 159

S2824 4 148 152 244 260 215 215 123 139 249 257 265 269 -9 -9 161 165 158 170 -9 -9 -9 -9 281 285 -9 -9 134 142 108 108 -9 -9 -9 -9

S2825 4 148 148 248 248 -9 -9 -9 -9 257 257 265 269 -9 -9 161 161 -9 -9 185 189 223 227 281 281 167 179 -9 -9 104 104 -9 -9 -9 -9

S2826 4 148 152 248 248 211 215 123 139 249 253 261 261 -9 -9 161 169 166 170 181 189 223 243 281 281 175 175 134 134 104 108 -9 -9 147 147

S2829 4 144 152 260 264 -9 -9 123 123 249 257 265 273 187 199 161 165 170 170 189 189 223 223 277 281 167 167 -9 -9 108 108 -9 -9 -9 -9

S2830 4 148 160 252 260 207 215 123 131 253 257 261 269 187 187 165 165 162 166 189 193 223 243 -9 -9 175 183 138 138 104 104 -9 -9 -9 -9

S2831 4 148 148 -9 -9 215 215 -9 -9 -9 -9 265 277 187 187 161 169 158 166 -9 -9 223 231 -9 -9 167 175 134 134 108 108 -9 -9 143 159

S2832 4 148 152 252 260 207 215 123 131 253 257 261 269 187 199 161 165 162 166 189 189 223 227 281 281 -9 -9 134 134 108 108 -9 -9 147 147

S2833 4 148 148 252 292 211 211 -9 -9 -9 -9 265 273 199 199 -9 -9 158 166 -9 -9 223 243 -9 -9 167 179 138 138 112 112 -9 -9 -9 -9

S2554 5 156 168 236 240 211 211 131 139 253 261 261 265 211 211 169 173 158 162 -9 -9 239 255 281 285 183 191 138 138 104 108 -9 -9 147 155

S2556 5 152 156 236 240 207 211 131 139 253 261 261 265 211 211 169 169 158 162 185 189 -9 -9 281 285 183 191 138 138 -9 -9 -9 -9 147 159

S2557 5 156 156 244 248 211 211 131 135 253 253 261 265 211 211 169 169 158 158 185 189 239 255 281 281 179 183 138 138 104 104 -9 -9 147 147

S2558 5 156 156 248 248 211 215 131 139 253 261 265 273 207 211 165 169 162 162 189 193 239 247 281 281 183 183 138 138 100 104 -9 -9 147 147

S2560 5 156 156 236 236 -9 -9 131 139 257 257 261 269 203 203 165 169 162 162 197 197 239 247 281 281 171 191 138 138 104 104 -9 -9 147 147

S2561 5 156 168 240 248 211 211 131 135 261 261 269 273 211 211 169 169 166 166 177 189 239 239 281 281 183 183 138 138 100 100 213 213 147 155

S2562 5 156 164 240 240 211 211 131 147 261 269 261 269 211 219 165 165 166 166 197 197 239 239 281 281 171 179 138 138 100 100 -9 -9 155 159

S2563 5 156 168 236 240 211 211 131 139 253 261 261 269 211 211 169 169 158 162 189 201 239 239 281 285 183 191 138 138 104 104 205 205 155 171

S2565 5 -9 -9 -9 -9 211 211 -9 -9 -9 -9 257 273 -9 -9 165 165 162 170 -9 -9 239 255 285 285 183 191 138 138 104 104 -9 -9 143 151

S2567 5 -9 -9 -9 -9 211 211 131 147 -9 -9 -9 -9 203 203 169 169 -9 -9 -9 -9 239 239 285 285 179 183 138 138 104 104 -9 -9 171 171

S2568 5 156 156 236 236 211 211 131 139 261 261 257 269 211 211 165 169 162 162 189 193 239 255 281 285 179 183 138 138 100 100 -9 -9 147 147

S2569 5 -9 -9 244 248 211 215 135 139 253 257 257 261 211 211 165 169 162 162 177 193 239 239 281 293 183 187 138 138 104 104 213 213 147 155

S2570 5 -9 -9 236 240 211 211 131 147 -9 -9 269 269 -9 -9 165 165 158 162 177 197 239 255 281 285 179 183 138 138 100 104 213 213 147 159

S2572 5 -9 -9 236 248 211 211 131 147 253 269 261 269 203 207 169 169 162 166 189 197 239 239 281 285 183 191 138 138 -9 -9 213 213 151 171

S2574 5 156 156 236 240 211 211 131 139 253 261 257 261 211 211 165 169 158 162 177 189 239 255 281 281 179 183 138 138 -9 -9 -9 -9 147 155

S2575 5 156 156 240 244 211 211 131 139 257 269 257 273 207 219 165 169 162 162 -9 -9 239 239 281 281 183 187 138 138 100 100 -9 -9 147 159

S2576 5 152 156 236 236 211 211 131 139 253 261 269 277 211 211 165 165 162 162 -9 -9 239 239 281 293 179 183 138 138 -9 -9 205 221 147 151

S2577 5 156 156 236 244 211 211 135 147 257 261 269 269 207 215 169 169 166 166 193 193 239 247 281 285 179 179 134 134 100 100 213 221 147 147

S2578 5 156 168 236 252 211 211 131 147 253 257 257 273 207 219 165 165 162 162 189 193 239 239 281 285 183 187 134 134 100 100 221 221 147 151

S2579 5 156 156 236 236 211 211 135 135 257 261 269 269 203 215 165 165 166 170 197 197 239 239 281 293 183 187 138 138 -9 -9 -9 -9 -9 -9

S2581 5 156 156 236 244 211 211 135 135 261 269 -9 -9 203 211 165 169 166 166 177 197 239 239 281 281 187 187 134 134 104 104 -9 -9 147 159

S2582 5 156 156 -9 -9 211 211 139 139 -9 -9 -9 -9 -9 -9 165 165 158 170 -9 -9 -9 -9 281 293 179 183 134 138 -9 -9 -9 -9 147 155

S2584 5 -9 -9 -9 -9 211 215 131 147 257 269 261 265 203 211 165 169 -9 -9 189 193 239 243 281 281 -9 -9 138 138 104 104 209 209 143 143

S2585 5 156 156 236 248 211 211 139 139 261 269 269 269 -9 -9 -9 -9 162 166 -9 -9 239 239 281 281 179 187 138 134 104 104 209 209 143 143

S2586 5 156 156 236 248 211 211 131 147 261 261 269 269 211 211 165 169 162 166 177 189 239 255 281 281 183 183 138 134 104 108 209 233 147 155

S2587 5 156 156 244 248 211 211 139 139 257 269 261 269 -9 -9 -9 -9 162 162 189 201 239 255 281 281 187 187 134 138 -9 -9 209 209 143 151

S2588 5 156 156 236 248 211 211 131 143 253 261 257 261 203 215 -9 -9 162 162 169 189 239 255 281 281 -9 -9 134 138 108 112 209 233 143 151

S2589 5 -9 -9 236 248 211 211 131 139 253 261 261 265 215 219 165 165 162 162 169 197 239 239 281 281 183 187 134 138 100 104 209 209 143 151

S2590 5 -9 -9 -9 -9 199 211 -9 -9 253 253 257 269 -9 -9 -9 -9 158 162 193 197 -9 -9 281 281 -9 -9 138 138 100 104 -9 -9 143 147

S2591 5 156 156 236 240 211 215 139 147 257 269 261 269 207 211 -9 -9 158 162 -9 -9 239 255 281 281 183 191 138 138 104 104 209 229 143 151

S2592 5 156 168 248 248 211 211 135 143 253 261 257 269 211 219 165 165 -9 -9 -9 -9 239 239 281 281 -9 -9 134 138 104 104 -9 -9 143 155

S2593 5 156 156 236 236 211 211 131 131 269 269 261 273 219 219 169 169 162 162 189 189 239 239 281 281 183 183 -9 -9 104 104 209 209 151 155

S2594 5 156 156 248 248 211 211 135 139 253 253 261 269 203 203 165 169 162 162 189 197 243 255 281 281 -9 -9 134 134 108 108 233 233 147 147

S2598 5 156 156 236 240 211 211 131 139 261 269 257 269 -9 -9 165 169 -9 -9 197 197 239 255 281 281 179 183 134 134 104 112 -9 -9 -9 -9

S2599 5 156 168 236 244 -9 -9 139 147 261 257 269 269 203 211 165 165 162 162 -9 -9 239 255 285 293 179 183 134 138 104 104 -9 -9 147 147

S2600 5 156 156 236 248 211 211 -9 -9 253 261 269 269 219 219 165 169 162 162 197 197 239 239 281 293 187 187 134 138 104 104 181 233 147 147

S2601 5 156 156 236 236 211 215 135 147 261 269 261 269 211 215 169 169 158 162 189 193 239 247 281 281 183 183 134 134 104 108 217 217 155 155

S2602 5 156 168 236 240 199 199 139 139 261 261 269 269 207 211 165 169 166 170 189 189 239 239 281 293 183 183 138 138 104 108 205 209 147 151

S2604 5 -9 -9 236 244 -9 -9 -9 -9 257 261 261 261 211 215 165 169 162 166 -9 -9 247 255 281 281 183 187 134 138 104 104 -9 -9 147 155

S2605 5 156 168 -9 -9 -9 -9 131 139 253 261 261 269 211 219 165 169 162 162 -9 -9 -9 -9 285 293 183 187 138 138 104 104 181 205 -9 -9

S2606 5 -9 -9 -9 -9 211 219 139 147 261 269 261 269 211 211 169 169 158 162 189 197 239 239 285 285 183 183 134 134 -9 -9 -9 -9 -9 -9

S2607 5 156 156 244 248 211 211 147 147 261 261 265 269 203 207 165 169 162 162 189 197 239 255 285 293 183 183 134 134 104 108 -9 -9 -9 -9

S2609 5 156 156 248 248 211 211 131 147 253 261 269 269 219 219 169 169 162 166 189 197 239 255 285 293 183 183 134 134 104 108 -9 -9 147 159

S2611 5 156 156 236 244 211 211 135 143 253 269 269 269 211 219 169 169 162 162 189 197 239 247 281 293 183 183 134 138 104 108 201 205 143 147

S2612 5 156 168 -9 -9 211 211 131 131 253 269 261 269 207 211 165 165 162 162 189 189 -9 -9 -9 -9 179 179 134 134 104 104 -9 -9 147 155

S2613 5 -9 -9 248 248 211 211 139 139 253 269 261 269 -9 -9 165 165 162 162 189 197 239 243 293 293 179 187 134 134 104 104 181 221 151 159

S2614 5 156 156 240 244 -9 -9 139 147 257 261 261 269 211 219 169 169 158 166 189 197 -9 -9 281 293 171 187 134 134 104 104 213 233 147 147

S2617 5 -9 -9 236 240 211 215 139 147 253 261 269 269 -9 -9 169 169 162 162 189 197 239 255 -9 -9 -9 -9 134 134 104 108 181 181 147 155

S2619 5 -9 -9 248 248 211 215 131 147 257 261 269 269 -9 -9 165 165 154 162 189 189 239 243 293 293 171 179 134 138 104 108 205 205 147 147

S2620 5 -9 -9 240 248 211 219 131 147 253 261 261 269 211 219 169 169 -9 -9 173 189 239 239 281 285 179 183 134 134 104 104 205 205 -9 -9

S2621 5 156 164 236 248 211 211 139 139 261 261 261 269 -9 -9 169 169 162 166 177 189 239 243 -9 -9 183 187 134 134 104 108 181 181 151 151

S2622 5 156 156 236 244 211 211 139 147 253 261 261 261 211 219 165 169 162 162 189 197 239 255 281 281 183 183 134 134 104 104 181 233 147 147

S2624 5 156 156 244 244 211 211 135 143 269 269 261 269 219 219 165 169 162 166 -9 -9 239 255 285 293 179 183 134 134 104 108 181 213 147 147

S2625 5 156 156 236 240 211 211 131 131 253 253 261 269 211 219 165 165 162 166 189 197 239 239 281 293 179 187 134 134 108 112 181 233 147 155

S2626 5 156 160 236 240 -9 -9 -9 -9 261 269 257 261 211 219 169 169 162 162 -9 -9 243 255 285 285 183 183 134 134 104 108 -9 -9 147 147

S2627 5 156 156 244 248 -9 -9 135 139 257 261 261 269 207 207 165 169 162 162 -9 -9 239 255 281 285 187 187 138 138 104 112 181 181 155 155

S2628 5 156 156 248 248 211 211 -9 -9 253 261 261 269 203 219 -9 -9 162 162 189 197 239 239 281 293 183 183 134 138 108 108 217 217 155 155

S2629 5 156 156 236 236 211 215 131 139 261 269 269 269 207 219 169 169 158 162 189 189 243 255 281 285 171 179 134 138 -9 -9 181 217 147 147

S2631 5 -9 -9 240 248 211 211 -9 -9 261 261 269 269 211 211 165 165 158 162 189 189 239 255 281 281 183 191 134 138 104 104 181 205 147 151

S2633 5 156 156 240 240 211 211 -9 -9 257 261 261 273 207 215 165 165 162 162 173 189 -9 -9 281 281 179 183 134 138 104 104 221 221 151 151

S2634 5 156 156 236 240 211 211 139 147 253 261 257 269 207 219 165 169 162 162 177 189 239 247 281 281 187 187 138 138 104 108 181 209 147 147

S2635 5 156 156 248 248 211 211 147 147 261 269 261 269 211 219 169 169 166 166 193 197 239 239 281 293 183 183 134 134 104 108 213 217 155 155

S2636 5 156 164 236 240 211 211 131 135 253 253 273 273 207 211 169 169 158 162 189 201 239 239 285 289 183 191 134 134 104 108 -9 -9 147 147

S2637 5 156 156 236 244 211 219 131 139 253 261 273 273 203 203 165 165 162 166 -9 -9 239 239 285 293 179 183 134 134 104 108 213 213 147 151

S2638 5 156 156 248 248 211 219 139 139 257 253 269 273 211 211 169 169 -9 -9 177 193 239 255 281 289 179 183 134 134 108 112 213 213 147 155

S2639 5 164 168 236 236 211 219 131 139 249 269 261 269 211 219 165 169 166 166 177 197 239 239 285 285 183 183 134 134 104 108 213 213 155 155

S2642 5 -9 -9 236 240 -9 -9 131 147 261 261 261 261 211 211 165 169 158 162 -9 -9 239 247 281 285 183 191 134 134 104 112 -9 -9 147 147
