# Supplementary material for: Genetic Structure and Evolutionary History of Rhinopithecus roxellana in Qinling Mountains, Central China
Source: Front Genet. 2021 Jan 20;11:611914. doi: 10.3389/fgene.2020.611914 (PMC7855588; doi:10.3389/fgene.2020.611914)

# Supplementary Tables

Table S1: Microsatellite marker information

| Locus | Primer sequence (5'-3') | *T*_A_ | Size | Fluo | Motif | *F*_IS_ | *P*-Val |
| --- | --- | --- | --- | --- | --- | --- | --- |
| *D10S1432* | Fwd:CAGTGGACACTAAACACAATCC Rev:TAGATTATCTAAATGGTGGATTTCC | 54 | 150 | Fam | TATC | -0.042 | 0.576 |
| *D10S2483* | Fwd:CAGGTTTTGCAATTGCTTTC Rev:AACCATCTCCACCCACATAA | 55 | 170 | Fam | GATA | 0.150 | 0.170 |
| *D10S676* | Fwd:GAGAACAGACCCCCAAATCT Rev:ATTTCAGTTTTACTATGTGCATGC | 61 | 210 | Tamra | GATA | 0.262 | 0.142 |
| *D12S375* | Fwd:TTGTTGAGGGTCTTTCTCCA Rev:TCTTCTTATTTGGAAAAGTAACCC | 60 | 180 | Tamra | TATC | -0.101 | 0.606 |
| *D14S306* | Fwd:AAAGCTACATCCAAATTAGGTAGG Rev:TGACAAAGAAACTAAAATGTCCC | 52 | 190 | Tamra | GATA | -0.089 | 0.076 |
| *D19S248* | Fwd:GTCCAAGGAGACAGAGCCA Rev:ACTGTGCCTGACTTCTGCT | 61 | 104 | Hex | GATA | -0.152 | 0.127 |
| *D19S582* | Fwd:TGTGAGCAGAGAGATGGACA Rev:ACAGTGAGTTTGATCTCTAGCA | 61 | 130 | Fam | TATC | -0.117 | 1.000 |
| *D21S2054* | Fwd:GCAGTAAATGTCTATGAAACAAGG Rev:ATGATAGGTAGATGGATCAATTAGA | 53 | 180 | Tamra | TATC | 0.034 | 0.201 |
| *D3S1766* | Fwd:ACCACATGAGCCAATTCTGT Rev:ACCCAATTATGGTGTTGTTACC | 60 | 230 | Hex | TATC | -0.160 | 0.368 |
| *D6S1036* | Fwd:ATCCCAACTCTTAAATGGGC Rev:TTCCATGGCAGAAATTGTTT | 53 | 260 | Tamra | TATC | 0.059 | 0.160 |
| *D6S1040* | Fwd:GAATGCAGGACTGTTTCTGG Rev:TATATTTCTTGGGAAAGATAGATGG | 60 | 220 | Tamra | TATC | 0.013 | 0.835 |
| *D6S501* | Fwd:GCTGGAAACTGATAAGGGCT Rev:GCCACCCTGGCTAAGTTACT | 58 | 160 | Fam | TATC | 0.016 | 0.827 |
| *D7S1804* | Fwd:TTCAAGTGGTTGGGTTCACT Rev:TGGGTCTAGTCCAGTGGTGT | 60 | 240 | Hex | TATC | 0.031 | 0.326 |
| *D7S2204* | Fwd:TCATGACAAAACAGAAATTAAGTG Rev:AGTAAATGGAATTGCTTGTTACC | 54 | 260 | Hex | TATC | -0.001 | 0.420 |
| *D7S820* | Fwd:ATGTTGGTCAGGCTGACTATG Rev:GATTCCACATTTATCCTCATTGAC | 61 | 250 | Hex | GATA | 0.088 | 0.862 |
| *D8S1049* | Fwd:TAAGTCAAACAAGCAAAGTGC Rev:CCTCCTATTGCTTTTTCCAAA | 55 | 140 | Fam | GATA | -0.071 | 0.731 |
| *D9S252* | Fwd:ACCATGATTTGTCAACTCCTA Rev:ACAATGAACATCCATATACCC | 56 | 220 | Tamra | GATA | 0.112 | 0.378 |
| *D9S905* | Fwd:GTGGGAAAATTGGCCTAAGT Rev:CTTCTGAGCCTCACACCTGT | 54 | 280 | Hex | ATTC | -0.061 | 0.095 |
| *TPOX* | Fwd:GCACAGAACAGGCACTTAGG Rev:CCAAAATTGAACTCCTCA | 61 | 230 | Hex | AATG | 0.034 | 0.530 |

* *T*_A_ denotes the annealing temperature of the primers (°C), size denotes the size of PCR products (bp), Fluo denotes the fluorescence label, *N* denotes the number of individuals types, *F*_IS_ denotes the Wright’s inbreeding coefficient, *P*-Val denotes the Hardy-Weinberg equilibrium test.

Table S2: Prior distributions for model parameters used in divergence model comparisons (Fig. 3a).

| Parameter | Scenario | | |
| --- | --- | --- | --- |
|  | Prior Distribution | Minimum | Maximum |
| Effective population size | | | |
| NA | uniform | 10 | 200000 |
| N1 | uniform | 10000 | 1000000 |
| N2 | uniform | 10000 | 2000000 |
| N3 | uniform | 10000 | 2000000 |
| Time of events | | | |
| t1 | uniform | 10 | 400000 |
| t2 | uniform | 10 | 1000000 |
| Mean mutation rate uniform | uniform | 1.0×10^-7^ | 1.0×10^-5^ |
| Individual locus mutation rate | Gamma | 1.0×10^-8^ | 1.0×10^-5^ |
| Mean coefficient *P* | uniform | 0.1 | 0.7 |
| Individual locus coefficient *P* | Gamma | 0.01 | 0.9 |

3 Table S3 the seven bioclimatic variables that were used in ecological niche modelling.

| Code | Bioclimatic variable |
| --- | --- |
| BIO2 | Mean Diurnal Range (Mean of monthly (max temp - min temp)) |
| BIO5 | Max Temperature of Warmest Month |
| BIO6 | Min Temperature of Coldest Month |
| BIO7 | Temperature Annual Range (BIO5-BIO6) |
| BIO14 | Precipitation of Driest Month |
| BIO15 | Precipitation Seasonality (Coefficient of Variation) |
| BIO18 | Precipitation of Warmest Quarter |

4 Figure S1 the posterior probabilties of the ten scenarios


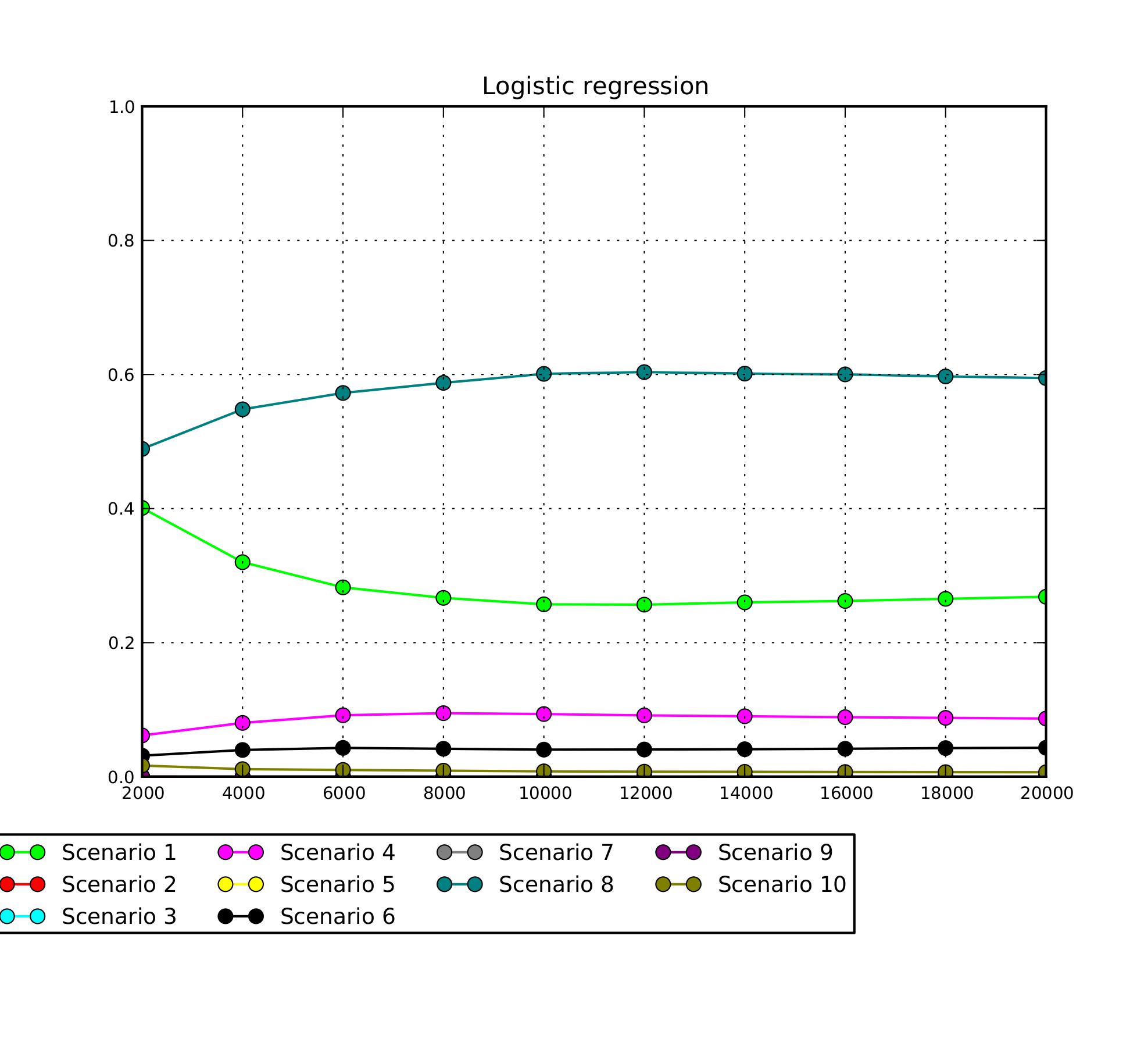

Supplement: Supplementary Figure 1 — The posterior probabilities of the 10 scenarios. [file Data_Sheet_1.docx]
